# Surface engineering of hierarchical platinum-cobalt nanowires for efficient electrocatalysis

**DOI:** 10.1038/ncomms11850

**Published:** 2016-06-29

**Authors:** Lingzheng Bu, Shaojun Guo, Xu Zhang, Xuan Shen, Dong Su, Gang Lu, Xing Zhu, Jianlin Yao, Jun Guo, Xiaoqing Huang

**Affiliations:** 1 College of Chemistry, Chemical Engineering and Materials Science, Soochow University, Jiangsu 215123, China; 2 Department of Materials Science and Engineering, College of Engineering, Peking University, Beijing 100871, China; 3 Department of Energy and Resources Engineering, College of Engineering, Peking University, Beijing 100871, China; 4 Department of Physics and Astronomy, California State University, Northridge 91330, California, USA; 5 Center for Functional Nanomaterials, Brookhaven National Laboratory, Upton, New York 11973, USA; 6 Testing and Analysis Center, Soochow University, Jiangsu 215123, China

## Abstract

Despite intense research in past decades, the lack of high-performance catalysts for fuel cell reactions remains a challenge in realizing fuel cell technologies for transportation applications. Here we report a facile strategy for synthesizing hierarchical platinum-cobalt nanowires with high-index, platinum-rich facets and ordered intermetallic structure. These structural features enable unprecedented performance for the oxygen reduction and alcohol oxidation reactions. The specific/mass activities of the platinum-cobalt nanowires for oxygen reduction reaction are 39.6/33.7 times higher than commercial Pt/C catalyst, respectively. Density functional theory simulations reveal that the active threefold hollow sites on the platinum-rich high-index facets provide an additional factor in enhancing oxygen reduction reaction activities. The nanowires are stable in the electrochemical conditions and also thermally stable. This work may represent a key step towards scalable production of high-performance platinum-based nanowires for applications in catalysis and energy conversion.

## Abstract (web summary)

Platinum-based nanowires are promising for fuel cell applications due to their high catalytic activity. Here the authors report on hierarchical platinum-cobalt nanowires with high-index facets showing specific/mass activities for oxygen reduction reaction 39.6/33.7 times higher than commercial Pt/C catalyst.

## Graphical abstract

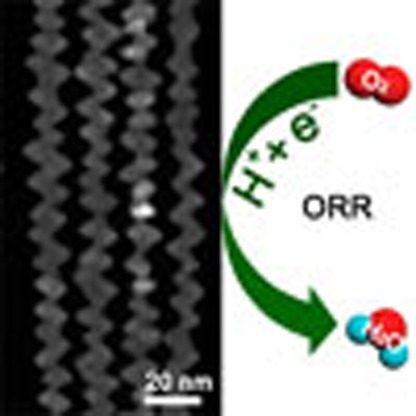

Fuel cells are highly desired devices for advanced portable electronic devices and electrical vehicles as they are promising candidates for providing a sustainable, clean and efficient energy supply^1^,^2^,^3^,^4^,^5^. The fuel cell catalyses the reactions between a fuel (methanol, ethanol and molecular hydrogen) at the anode and molecular oxygen at the cathode, converting chemical energy into electrical power via the electrochemical process^6^,^7^,^8^. Catalysts are the central components of fuel cell technologies and largely dictate their ultimate performance. To date, Pt-based nanomaterials are recognized as the most effective electrocatalysts for both the anodic oxidation reaction and the cathodic oxygen reduction reaction (ORR) in the fuel cells^9^,^10^,^11^. Currently, the state-of-the-art electrocatalysts are almost exclusively small platinum (Pt) nanoparticles (NPs) supported on carbon black (Pt/C). However, the practical large-scale commercialization of fuel cells is still a challenge because of the high cost, poor reaction kinetics, loss of electrochemical surface area and catalytic activity over long-term duration of commercial Pt catalysts^12^,^13^,^14^,^15^,^16^.

Precise control over material structures at the nanoscale allows for command over both surface structure and composition, thereby imparting enhanced and new functionality^17^,^18^,^19^,^20^. In the search for high-performance electrocatalysts^9^,^10^,^21^,^22^,^23^,^24^, one-dimensional (1D) Pt alloy nanostructures represent a promising direction for advanced catalysis because they possess multiple advantages, such as inherent anisotropic morphology, high flexibility, high surface area and high conductivity, compared with their 0D counterparts, making them work more effectively for ORR activity and durability enhancement than traditional Pt alloy NPs^25^,^26^. This is because during the catalysis process, individual nanowires (NWs) can have a higher surface area interacting with carbon support than NPs, which not only enhances electron transfer between oxygen and the Pt surface, but also facilitates bonding between the NWs and the carbon support, rendering high stability. These advantages have stimulated explorations of new synthetic routes to create various Pt-*M* (*M*=iron (Fe)-, cobalt (Co)-, nickel (Ni))-based NWs, but unfortunately usually leading to conventional large-diameter Pt*M*-based NWs with smooth surfaces (lacking the strong electron effect), which are, however, not beneficial for creating more active sites for maximizing electrocatalytic performance, and ultrathin Pt-*M* NWs with limited durability because of their relatively small diameters^27^,^28^. Therefore, the biggest issue in using Pt-*M* NWs for ORR is that there is always a trade-off between activity and durability, making them impractical for fuel cell devices. In this regard, the creation of a new class of Pt*M* NWs with improved utilization of Pt atoms for simultaneous high catalytic activity and durability is highly desirable but remains a significant challenge.

Herein, we report the synthesis of a class of hierarchical Pt-Co NWs enclosed with high-density high-index facts through a robust large-scalable wet-chemical approach as electrocatalysts for fuel cell reactions. Unlike conventional Pt-*M* NWs, these hierarchical Pt-Co NWs with highly uneven surfaces are tailored to have an ordered intermetallic structure, high-index facets and a Pt-rich surface, which can meet several critical design criteria for efficient nanoelectrocatalysts^29^. They are fully functional for both high-performance anodic and cathodic fuel cell reactions, such as methanol oxidation reaction (MOR), ethanol oxidation reaction (EOR) and ORR that largely outperform those based on the commercial Pt/C catalyst. In particular, they deliver unprecedented ORR performance with the promising specific and mass activities of 7.12 mA cm^−2^ and 3.71 A mg^−1^_Pt_ at 0.9 V versus reversible hydrogen electrode (RHE), which are 39.6 and 33.7 times higher than those of the state-of-the-art Pt/C catalyst (Johnson Matthey, 20 wt% Pt, 0.18 mA cm^−2^ and 0.11 A mg^−1^_Pt_), making the hierarchical Pt-Co NWs the most active electrocatalyst among the Pt-Co-based nanomaterials for ORR reported to date, to the best of our knowledge^30^. Moreover, the hierarchical Pt-Co NWs show superior cycling stability in the ORR condition with negligible activity decay over the course of 20,000 cycles and are also thermally stable with no morphology change after annealing up to 500 °C. Their high thermal stability may provide a catalytic platform for high-temperature catalytic reactions, which is not easily achieved by particulate nanostructures because of their deformation or aggregation during the annealing treatment^31^,^32^. These hierarchical Pt-Co NWs enclosed with high-index facets enabled by our colloidal chemistry technique reported herein are promising catalyst candidates with unprecedented catalytic activity and stability for practical proton exchange membrane fuel cells.

## Results

### Synthesis and characterization of hierarchical Pt-Co NWs

The wet-chemical approach to produce the hierarchical Pt-Co NWs involves the use of platinum(II) acetylacetonate (Pt(acac)_2_) and cobalt(III) acetylacetonate (Co(acac)_3_) as the metal precursors, oleylamine as solvent and surfactant, cetyltrimethylammonium chloride (CTAC) as the structure-directing agent, and glucose as a reducing agent. Briefly, Pt(acac)_2_, Co(acac)_3_, oleylamine, glucose and CTAC in the controlled molar ratios were added into a 35-ml glass vial. After the glass vial had been capped, the mixture was fully ultrasonicated for around 1.5 h, heated from room temperature to 160 °C in 0.5 h, then kept at 160 °C for 8 h in an oil bath, and finally naturally cooled to room temperature (see the Methods section for details). The resulting black colloidal products were subjected to centrifugation and washed three times with ethanol/cyclohexane (v:v, 9:1) mixture. A variety of synthetic parameters, such as precursor, reducing agent and surfactant, were carried out for studying the optimal conditions for synthesizing well-defined Pt-Co NWs. The results show that the successful preparation of hierarchical Pt-Co NWs is highly controlled by the amount of CTAC, the select of Co(acac)_3_ and the use of glucose (Supplementary Figs 1–4).

**Figure 1:**
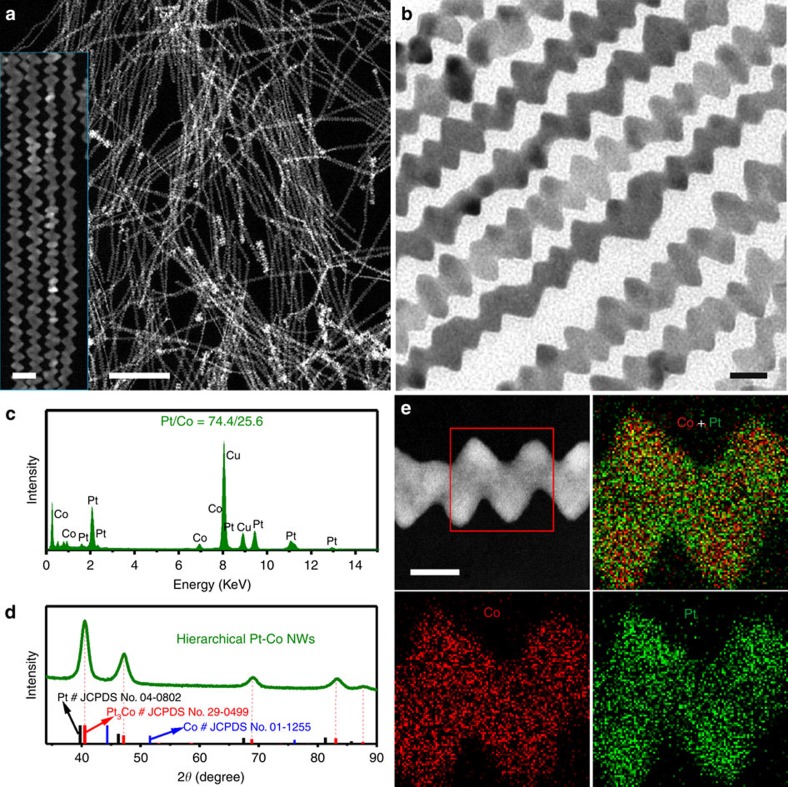
Morphology and structure characterization of hierarchical Pt-Co nanowires. Representative (**a**) STEM image, (**b**) TEM image, (**c**) TEM-EDS, (**d**) PXRD pattern and (**e**) STEM-ADF image and EDS elemental mappings of the hierarchical Pt_3_Co NWs. Inset in **a** is an enlarged STEM image. The composition is Pt/Co=74.8/25.2, as revealed by ICP-AES. The scale bars in **a**, inset of **a**, **b** and **e** are 200, 20, 10 and 10 nm, respectively.

Transmission electron microscopy (TEM) and high-angle annular dark-field scanning TEM (HAADF-STEM) were used to characterize the morphology of the as-prepared hierarchical Pt-Co NWs. The TEM and STEM images show that the product consists of abundant nanostructure with 1D contour at the first glance (Fig. 1 and Supplementary Fig. 5). The NWs were the dominant product with the typical yield over 90%. The length of the NWs is in the range of several micrometres and the average aspect ratio is ∼50. Strikingly, high-magnification HAADF-STEM (inset in Fig. 1a) and TEM images (Fig. 1b) reveal that each NW has a highly uneven diameter along their entire length, exhibiting highly uneven and the crenel-like hierarchical nanostructure. The overall Pt/Co composition is quantitatively determined to be 74.8/25.2 (Pt_3_Co), measured by inductively coupled plasma atomic emission spectroscopy (ICP-AES) and TEM energy-dispersive X-ray spectroscopy (TEM-EDS; Fig. 1c). The surface molar ratio of Pt to Co obtained from X-ray photoelectron spectroscopy (XPS) is 95.2/4.8, much higher than that of overall Pt/Co composition, revealing the Pt-rich surface structure (Supplementary Fig. 6). The powder X-ray diffraction (PXRD) pattern of the hierarchical Pt-Co NWs (Fig. 1d) displays the distinct facet-centred cubic structure associated with the alloyed Pt_3_Co (Pt_3_Co, JCPDS No. 29-0499). The alloyed structure was also confirmed by the STEM-EDS elemental mapping analysis, where the distributions of Pt and Co are even through the whole NW, despite the hierarchical Pt_3_Co NWs having very uneven surfaces (Fig. 1e and Supplementary Fig. 7).

**Figure 2:**
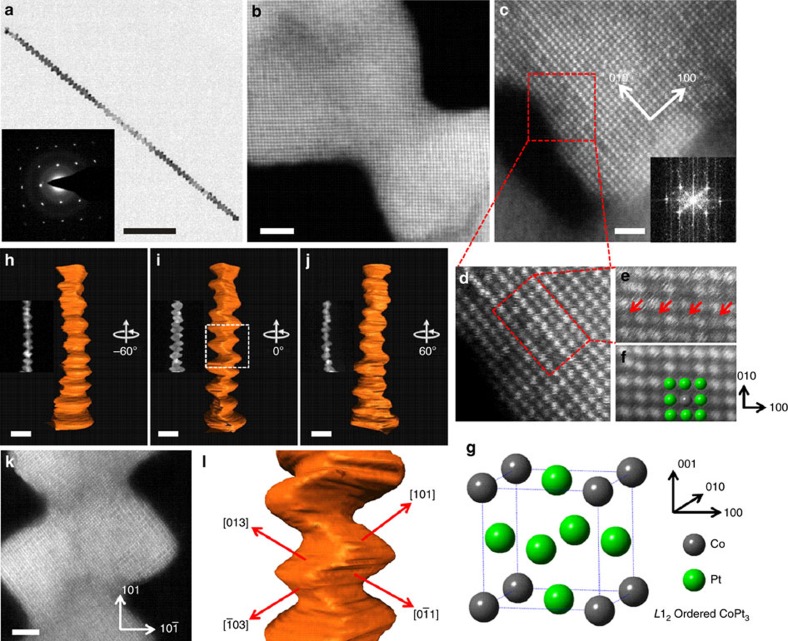
Structure analysis of hierarchical Pt-Co nanowires. (**a**) TEM image of an individual hierarchical Pt_3_Co NW. The inset shows the corresponding selected-area electron diffraction pattern. (**b**,**c**) Atomic-resolution HAADF-STEM images of Pt_3_Co NWs along [001] zone axis. The inset in (**c**) shows the corresponding Fast Fourier Transform (FFT) pattern. (**d**,**e**)The enlarged HAADF-STEM images from the region with *L*1_2_-ordered intermetallic structure. (**f**) FFT-filtered HAADF-STEM image from the same area of **e**. (**g**) Schematic diagram of the *L*1_2_-ordered intermetallic structure of Pt_3_Co. (**h**–**j**) A series of reconstructed 3D tomograms at viewing angles of −60^o^ (**h**), 0^o^ (**i**) and 60^o^ (**j**), the as-acquired HAADF-STEM images are shown in the insets. (**k**) An atomic resolution HAADF-STEM image at the viewing angles of 0^o^, and (**l**) indexes of the flat planes of the Pt_3_Co NWs. The scale bars in **a**–**c**, **h**–**k** are 100, 2, 1, 10, 10, 10 and 2 nm, respectively.

The hierarchical Pt_3_Co NWs are single crystalline, revealed by both selected-area electron diffraction and atomic resolution HAADF-STEM measurements (Fig. 2a,b and Supplementary Fig. 7). To identify the unconventional NWs, a number of techniques was utilized to further analyse the structure of hierarchical Pt_3_Co NWs. Figure 2c is an atomic-resolution HAADF-STEM image along the [001] zone axis. The local ordering is observed, corresponding to the *L*1_2_-ordered intermetallic structure of Pt_3_Co. The projected *L*1_2_ unit cell is composed of a periodic square array of pure Co columns surrounded by Pt columns at the face centres and corners of each unit cell (Fig. 2g). As the contrast of HAADF-STEM image is proportional to the average atomic numbers of the columns (Z-contrast),^33^ the Pt columns have higher brightness than that of the Co columns in Fig. 2d–f. This is different from the homogeneous alloyed phase, which has all columns with equal intensity and without the super periodicity. From the structural perspective, the ordered phases with definite composition and structure can provide the predictable control over structural, geometric and electronic effects for catalysis optimization, which cannot be afforded by the widely created conventional alloys.^30^ In order to identify the crystal indices of the flat planes in the hierarchical Pt_3_Co NWs, we applied a HAADF-STEM electron tomography technique to visualize the three-dimensional (3D) structure of the hierarchical Pt_3_Co NWs. The HAADF-STEM images were acquired from −65^o^ to 65^o^ with the intervals of 1^o^. Figure 2h–j and the inset show the 3D reconstructed tomograms of an individual hierarchical Pt_3_Co NW at −60^o^, 0^o^ and 60^o^ and corresponding HAADF-STEM images. Combined the results from electron tomography with those from the atomic resolution HAADF-STEM image shown in Fig. 2k, we found the [001] plane of the NWs aligns very close to the grid plane. Furthermore, by analysis of the cross-sectional structure along <101> direction of Pt_3_Co NWs, we could deduce the indexes of the flat planes as [310] and [110] (Fig. 2l). As the hierarchical Pt_3_Co NWs contain many uneven surfaces, high-density [310], high-index facets will be introduced onto individual NWs, which is the key to achieve high electrocatalyic activity.

### Growth mechanism

**Figure 3:**
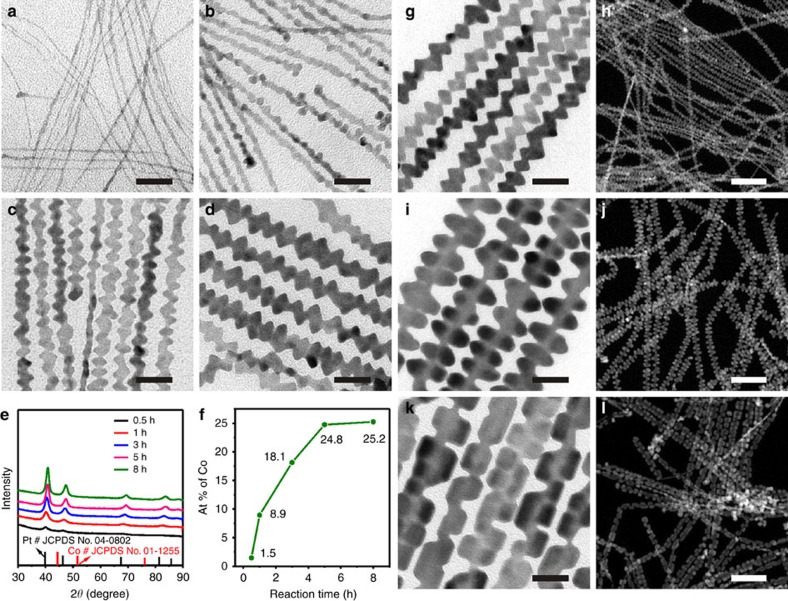
Growth mechanism and structural tuning. Typical TEM images of hierarchical Pt_3_Co NWs intermediates collected from (**a**) 0.5, (**b**) 1, (**c**) 3 and (**d**) 5 h. (**e**) PXRD patterns of hierarchical Pt_3_Co NWs intermediates collected from the reactions at different reaction times. (**f**) The composition ratio changes of Co to Pt for the hierarchical Pt_3_Co NWs intermediates, as determined by ICP-AES measurements. Typical TEM images and HAADF-STEM images of (**g**–**h**) hierarchical Pt_3_Co NWs, (**i**,**j**) hierarchical Pt_7_Co NWs and (**k**,**l**) hierarchical Pt_10_Co NWs collected from 8 h reactions. The scale bars in **a**–**d**, **g**, **i** and **k** are 20 nm. The scale bars in **h**,**j** and **l** are 100 nm.

The ability to make hierarchical Pt_3_Co NWs with unconventional surface structures is the most striking feature of our approach. 1D Pt-*M* nanostructures with such features have not been achieved previously, to the best of our knowledge. To understand their growth mechanism, the intermediates collected at different growth durations were carefully investigated (Fig. 3a–h). The TEM results show that ultrathin NWs were already formed at the early stage of the synthesis (30 min, Fig. 3a). These ultrathin NWs are of essentially pure Pt nanomaterials, as revealed by the PXRD and energy dispersive X-ray spectroscopy analyses (Fig. 3e,f). The diameter of ultrathin NWs is measured to be around 2 nm. As the reaction time was prolonged to 1 h, the surface of the NWs became more uneven (Fig. 3b), along with the average diameter increased from 2 to 5 nm. 8.9% Co was detected in the NWs of 5 nm (Fig. 3f). Together with the increase of the diameter in the NWs, further increase in the Co content and the presence of obvious humps in the intermediates were observed after 3.0 h (Fig. 3c,f). When the reaction time was 5.0 h, typical hierarchical PtCo NWs were obtained (Fig. 3d) with the Pt/Co ratio of about 3/1 (Fig. 3f). When the reaction time was increased to 8 h, there was no significant change in the diameter and morphology of NWs (Fig. 3g,h). These time-dependent structure and composition changes reveal that the formation of such hierarchical Pt_3_Co NWs relies upon the initial formation of ultrathin Pt NWs, the reduction of Co species onto the preformed NWs, and hereafter the inter-diffusion to form alloyed Pt_3_Co NWs (Fig. 3e,f). As time goes on, the diffraction peaks shift towards higher 2*θ* values with the increase of Co content (Fig. 3e). During the reaction time from 1.0 to 3.0 h, both the peak positions in the PXRD patterns and the structure of the NWs in unevenness change obviously. High-resolution TEM (HRTEM) and elemental mappings of the hierarchical Pt-Co NW intermediates at different growth times were also carried out. As shown in the HRTEM images (Supplementary Fig. 8), as the reaction proceeds, the humps on the surface of the preformed Pt NWs gradually evolve. As revealed by the HAADF-STEM elemental mappings, the Pt and Co distributions of each hierarchical Pt-Co NW intermediates are even throughout the whole growth process, despite the contents of Co increase as the reaction time prolonged (Supplementary Fig. 9). As the presence of very clear humps is coincident with the large increase of Co content in the Pt_3_Co NWs, we infer that the different reduction potentials and diffusions of Pt and Co should be the major driving force for the creation of such hierarchical NWs in the present condition, being consistent with the synthesis of the unconventional silicon NWs with periodic shell, where the diffusion of silicon adatoms is believed to be the most significant kinetic factor in controlling the growth of periodic shell.^34^

These TEM and composition studies as a function of reaction time indicate that the amount of Co(acac)_3_ introduced can significantly affect the growth of the hierarchical Pt-Co NWs. As shown in Supplementary Fig. 4a,b, the conventional NWs rather than hierarchical ones were obtained when the synthesis was carried out in the absence of Co(acac)_3_, whereas the introduction of Co(acac)_3_ could greatly impact the growth of NWs (Fig. 3i–l). If the amount of Co(acac)_3_ was reduced from 9.0 to 3.0 and 1.5 mg, the diameter of the obtained hierarchical Pt-Co NWs changed from 12 to 18 and 21 nm, associated with the reduced density of bumps (Fig. 3i–l and Supplementary Figs 10–16). When the amount of Co(acac)_3_ was increased to 12.0 mg, the diameter and structure of hierarchical Pt-Co NWs were almost unchanged (Supplementary Fig. 4c,d). The hierarchical Pt-Co NWs were yielded once Co(acac)_3_ was fed, although the hierarchical feature was highly depended on the amount of Co(acac)_3_ introduced. These results might be caused by the amount of Co(acac)_3_, which could dictate the reduction/diffusion behaviour of Co during the growth, and thus control the hierarchical degree of the Pt-Co NWs.^34^

### MOR and EOR performance

**Figure 4:**
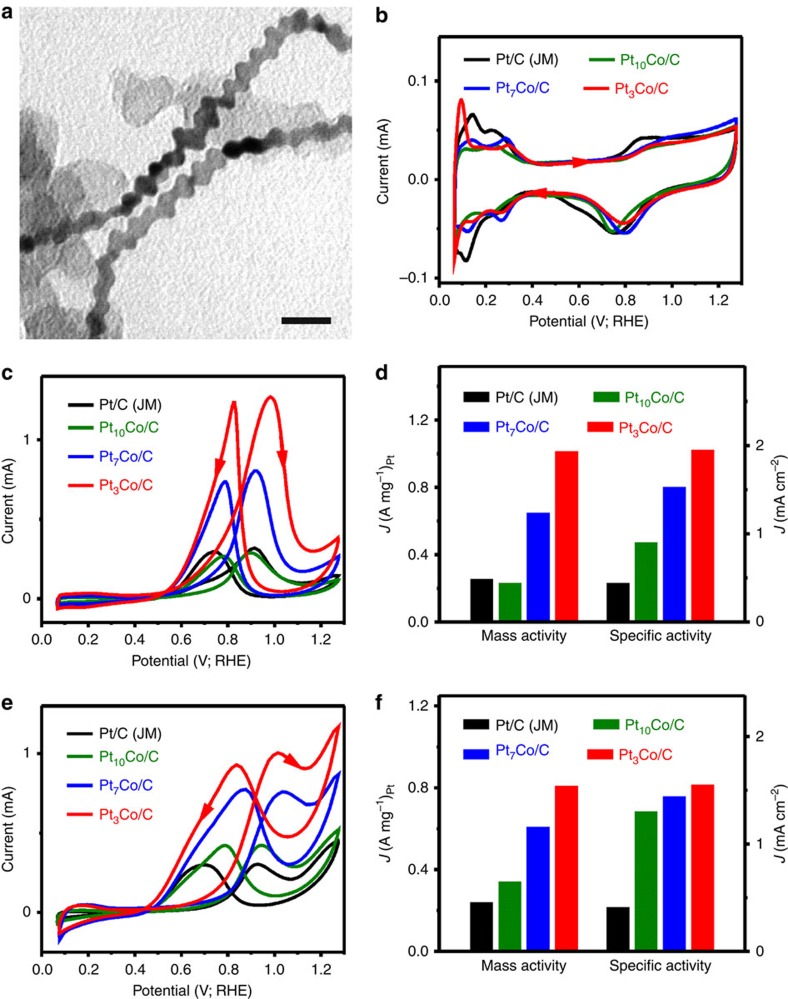
Alcohol electro-oxidation performance comparison. (**a**) Representative TEM image and (**b**) CVs of different catalysts in 0.1 M HClO_4_ solution. (**c**) CVs and (**d**) histogram of mass and specific activities of different catalysts for MOR in 0.1 M HClO_4_+0.2 M methanol solution. (**e**) CVs and (**f**) histogram of mass and specific activities of different catalysts for EOR in 0.1 M HClO_4_+0.2 M ethanol solution. The scale bar in **a** is 20 nm.

The hierarchical Pt-Co NWs were loaded on a commercial carbon (C, Vulcan) support by simply sonicating the Pt-Co NWs and C in cyclohexane, then washed with an ethanol/cyclohexane mixture and an ethanol/acetic acid mixture. Through such treatments, the hierarchical Pt-Co NWs were uniformly deposited onto carbon support with slightly composition changes from Pt_74.8_Co_25.2_ to Pt_75.5_Co_24.5_, while retaining their original nanostructures (Fig. 4a and Supplementary Figs 17 and 18). The limited loss of the Co was mainly caused by the formation of Pt-rich surface after this treatment, as confirmed by XPS characterization (Supplementary Fig. 19). Once the Pt-rich surface is present as a thin shell in a core/shell structure, both its activity and durability can be greatly enhanced as a result of the simultaneous downshift of its d-band centre and surface strain induced in the Pt surface^25^,^27^.

The resulting hierarchical Pt-Co NWs catalysts (Pt-Co NWs/C) were initially evaluated as anodic oxidation electrocatalysts for both MOR and EOR. To this end, three hierarchical PtCo NWs/C with distinct compositions, namely Pt_3_Co NWs/C (Supplementary Figs 5–7 and 17), Pt_7_Co NWs/C (Supplementary Figs 11–13 and 17) and Pt_10_Co NWs/C (Supplementary Figs 14–16 and 17), were selected, and benchmarked against commercial Pt/C from JM (Pt/C, 20 wt% Pt on Vulcan XC72R carbon, Pt particle size: 2–5 nm, Supplementary Fig. 20). The catalysts were dispersed in a mixture of isopropanol/water/Nafion, and then drop onto glassy carbon electrode. The loading amount of Pt was kept to be 1.25 μg for all the catalysts. The cyclic voltammograms (CVs) of different electrocatalysts in N_2_-purged 0.1 M perchloric acid solution at a sweep rate of 50 mV s^−1^ are shown in Fig. 4b. The current responses from hydrogen adsorption/desorption processes appear in the potential range of 0.05–0.35 V. The electrochemically active surface areas (ECSAs) of Pt_3_Co NWs/C, Pt_7_Co NWs/C, Pt_10_Co NWs/C, Pt_3_Co NPs/C and Pt/C catalysts were calculated to be 52.1, 42.3, 25.8, 38.7 and 58.8 m^2^ g^−1^, respectively. Among all the Pt-Co NWs/C catalysts, the Pt_3_Co NWs/C catalysts display the highest ECSA of 52.1 m^2^ g^−1^ because of the most high-density dendritic structure, comparable to that of the commercial Pt/C (58.8 m^2^ g^−1^) catalyst.

The MOR measurements were carried out in 0.1 M HClO_4_+0.1 M CH_3_OH solutions at a sweep rate of 50 mV s^−1^. Figure 4c compares the CVs of three different Pt-Co NWs/C and commercial Pt/C for methanol electrooxidation. The mass and specific activities of all the catalysts are presented in Fig. 4d, in which the Pt_3_Co NWs/C shows the highest mass activity and specific activity. The peak current densities in the forward (positive) potential scan were 1.95, 1.53, 0.90 and 0.44 mA cm^−2^ on the Pt_3_Co NWs/C, Pt_7_Co NWs/C, Pt_10_Co NWs/C and Pt/C catalyst, respectively. The maximum mass activity of Pt_3_Co NWs/C is measured to be 1.02 A mg^−1^_Pt_, which is 1.59, 4.43 and 4.08 times higher than those of the Pt_7_Co NWs/C, Pt_10_Co NWs/C and Pt/C catalysts, respectively (Fig. 4d). We also evaluated the electrocatalytic activities of the Pt-Co NWs/C catalysts for EOR. As expected, all the Pt-Co NWs/C catalysts exhibit excellent electrocatalytic activity towards EOR (Fig. 4e), in which Pt_3_Co NWs/C has the highest activities by displaying excellent specific activity of 1.55 mA cm^−2^ and high mass activity of 0.81 A mg^−1^_Pt_, which are 3.78 and 3.37 times higher than those of commercial Pt/C, respectively (Fig. 4f), and higher than those of a home-made Pt_3_Co NPs/C (Supplementary Figs 21 and 22 and Supplementary Tables 1 and 2).

### ORR performance

**Figure 5:**
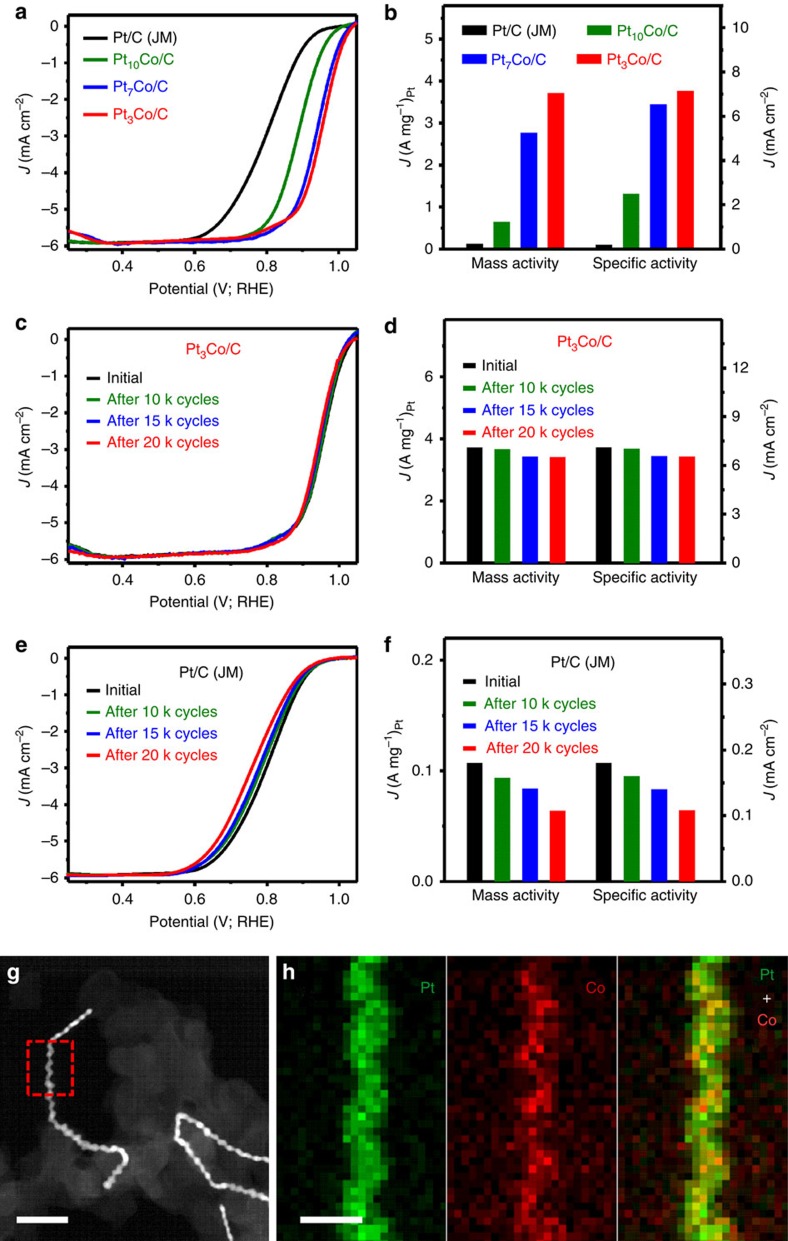
ORR performance comparison. (**a**) ORR polarization curves and (**b**) ORR-specific activities and mass activities of different catalysts. The ORR polarization curves were recorded at room temperature in an O_2_-saturated 0.1 M HClO_4_ aqueous solution at a sweep rate of 10 mV s^−1^ and a rotation rate of 1,600 r.p.m. (**c**) ORR polarization curves of the hierarchical Pt_3_Co NWs/C catalyst before and after 10,000, 15,000 and 20,000 potential cycles between 0.6 and 1.1 V versus RHE. (**d**) The changes on specific activities and mass activities of the hierarchical Pt_3_Co NWs/C catalyst before and after 10,000, 15,000 and 20,000 potential cycles. (**e**) ORR polarization curves of the commercial Pt/C catalyst before and after 10,000, 15,000 and 20,000 potential cycles between 0.6 and 1.1 V versus RHE. (**f**) The changes on specific activities and mass activities of the commercial Pt/C catalyst before and after 10,000, 15,000 and 20,000 potential cycles. The durability tests were carried out at room temperature in 0.1 M HClO_4_ at a scan rate of 100 mV s^−1^. (**g**) HAADF-STEM image and (**h**) elemental mappings of the hierarchical Pt_3_Co NWs/C after 20,000 cycles. The scale bars in **g** and **h** are 50 and 10 nm, respectively. The elemental mapping in **h** comes from the red-dotted line-marked area in **g**.

With their unique structural features, the Pt-Co NWs/C catalysts were further evaluated as cathodic electrocatalysts for ORR. Figure 5a shows the ORR polarization curves of the different catalysts recorded at room temperature in an O_2_-saturated 0.1 M HClO_4_ solution. The kinetic currents at 0.9 V versus RHE were normalized to ECSA and the mass loading of Pt to obtain the specific and mass activities, respectively. Among three kinds of Pt-Co NWs/C investigated, Pt_3_Co NWs/C has the highest specific activity of 7.12 mA cm^−2^ at 0.9 V versus RHE (Fig. 5b) with an impressive improvement factor of 39.6 over commercial Pt/C catalyst. Its mass activity reaches to 3.71 A mg^−1^_Pt_, 33.7-fold higher than that of commercial Pt/C catalyst. Although the Pt_3_Co NPs/C catalyst has the same composition with the Pt_3_Co NWs/C catalyst, the ORR activities of the Pt_3_Co NPs/C catalyst are much lower than the Pt_3_Co NWs/C catalyst (Fig. 5 and Supplementary Fig. 22), further displaying the super-high performance for ORR of the Pt_3_Co NWs/C catalyst (Supplementary Table 3). Those results make the Pt_3_Co NWs/C the most efficient catalyst ever achieved in the Pt-Co-based ORR catalysts to date to the best of our knowledge^30^, whose specific and mass activities are higher than those of the recent Pt-based nanocatalysts.^35^,^36^,^37^,^38^

The hierarchical Pt-Co NWs/C catalyst also shows superior electrochemical stability, accessed by using an accelerated durability test between 0.6 and 1.1 V (versus RHE) in 0.1 M HClO_4_ at a scan rate of 100 mV s^−1^. Figure 5c,d shows the ORR polarization curves of the hierarchical Pt_3_Co NWs/C catalyst before and after 10,000, 15,000 and 20,000 potential cycles. After 20,000 potential cycles, the activity of the hierarchical Pt_3_Co NWs/C catalyst is still as high as 3.41 A mg^−1^_Pt_ (91.9% of the initial value), which is 31-fold higher than that of the commercial Pt/C catalyst. The morphology and composition of hierarchical Pt_3_Co NWs/C catalyst after 20,000 electrochemical cycles was thoroughly studied (Fig. 5g,h and Supplementary Figs 23 and 24), showing there is no morphology (Fig. 5g and Supplementary Figs 23 and 24), alloyed structure (Fig. 5h and Supplementary Fig. 24) and composition changes (Supplementary Fig. 23) on the hierarchical Pt-Co NWs after 20,000 potential cycles. For a comparison, only ∼60% of initial activity could be retained (Fig. 5e,f), and serious NP aggregation was observed for the commercial Pt/C catalyst after the identical durability tests (Supplementary Fig. 20c,d). All these data indicate that hierarchical Pt-Co NWs are stable for ORR in strong acid solution.

### Theoretical investigation

**Figure 6:**
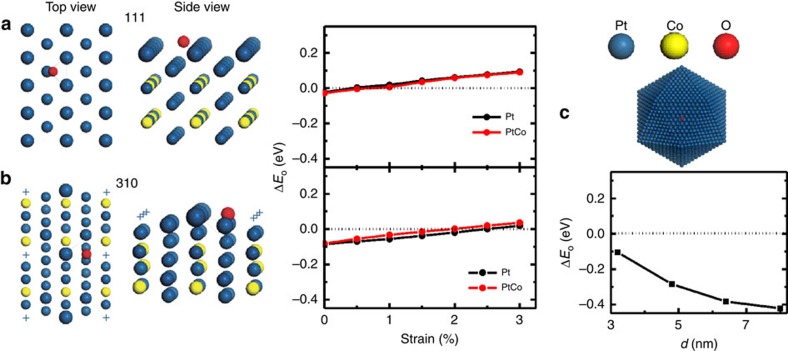
DFT calculations of oxygen adsorption energy. (**a**) Atomistic model of [110] surface and Δ*E*_O_ as a function of compressive strain. (**b**) Atomistic model of the MRR-[310] surface and Δ*E*_O_ as a function of compressive strain. Blue cross represents the missing atomic row on the top layer of [310] surface. The black and red curves correspond to Δ*E*_O_ values on the pure Pt and Pt_3_Co/Pt surface. (**c**) Δ*E*_O_ on [111] facet of the Pt NPs as a function of the particle size. The NP was modelled by an icosahedron with 20 [111] facets. The horizontal dashed line indicates the optimal Δ*E*_O_ value.

To shed light on the high-ORR performance on the hierarchical PtCo NWs, we performed density functional theory (DFT) calculations for the oxygen adsorption energy (*E*_O_) on the same high-index NW facets as observed in the HAADF-STEM measurements. *E*_O_ is widely used as a descriptor for ORR activities, and there exists an optimal value of *E*o, for which the ORR activity reaches the maximum^39^,^40^. For convenience, this optimal *E*_O_ was shift to 0 eV, thus Δ*E*_O_ represented the difference of a given *E*_O_ value relative to this optimal reference. It is known that the surface Co atoms in the PtCo NWs could dissolve under the electrochemical conditions, and be etched away, leading to core/shell-like structures with pure Pt as the shell. For such core/shell structures, in general, both surface strain and ligand effect could influence their catalytic activities, which are the subject of our first-principles simulations. Two typical adsorption sites, a bridge site at the edge of the NW and a hollow site on the facet of the NW, were examined on three surfaces, including [110], [310] and (2 × 1) missing-row reconstructed (MRR)-[310]. To examine the ligand effect on each of the three surfaces, we compared the pure Pt surface and the Pt_3_Co alloy surface with the top-layer Co atoms completely etched away (Pt_3_Co/Pt). To examine the strain effect, we calculated Δ*E*_O_ as a function of a compressive strain, ranging from 0 to 3%. The results of Δ*E*_O_ are shown in Fig. 6a,b and Supplementary Fig. 25. Moreover, we also calculated Δ*E*_O_ on the [111] facet of Pt NPs with the particle diameter ranging from 3 to 8 nm using a combined quantum mechanics-molecular mechanics method,^41^,^42^ and the results are shown in Fig. 6c. First, we found that the threefold hollow sites on both [110] and MRR-[310] surfaces were highly active for ORR. Their Δ*E*_O_ values were much closer to zero than that of Pt NPs, suggesting that the threefold hollow sites on the NWs could offer superior ORR activities to the Pt NPs. This similar enhancement in ORR activities has also been observed on the threefold hollow sites of high-index [211] surfaces calculated by Norskov *et al*.^43^ Hence, the additionally enhanced ORR activities in our Pt_3_Co NWs can be attributed to the threefold hollow sites on the high-index facets. Second, Δ*E*_O_ was found to increase linearly as a function of the compressive strain on both [110] and MRR-[310] surfaces, as shown in Fig. 6a,b. The compressive strain could relieve the over-binding of oxygen to the surfaces, thus enhance the ORR activities. In contrast, the ligand effect was negligible as the Δ*E*_O_ values on the pure Pt and the Pt_3_Co/Pt surfaces were almost the same. Therefore, the further improved ORR activities on the high-index faceted PtCo NWs stem from: (i) the active threefold hollow sites and (ii) the compressive strain on the Pt surface owing to the smaller atomic size of Co.

### Anti-aggregation properties

Another significant feature of the hierarchical Pt-Co NWs is that they can exhibit excellent anti-aggregation properties upon the high-temperature annealing treatment. As shown in Supplementary Fig. 26, the hierarchical Pt_3_Co NWs can be thermally stable up to 500 °C, as the 1D hierarchical structure was largely maintained after they were annealed at 500 °C for 1 h in N_2_ atmosphere. The high thermal stability of the hierarchical Pt_3_Co NWs is beneficial for the design of catalytic systems for high-temperature reactions or chemical processes, which is not easily achieved by the particulate Pt nanocrystals because of their poor anti-deformation ability.

## Discussion

To summarize, we demonstrate a large-scalable, wet-chemical approach to synthesize a class of Pt-Co NWs consisting of large-density high-index facets and Pt-rich surfaces. These features enable them to exhibit impressive activity and durability towards both the alcohol oxidations and ORR, to satisfy requirements for efficient electrocatalytic applications. They show high specific and mass activities for both MOR and EOR, largely outperforming those based on the commercial Pt/C catalyst. For the cathodic ORR, they can deliver unprecedented activities that are higher than the best Pt-Co-based catalysts for ORR, representing the highest activities ever achieved in the Pt-Co-based ORR catalysts, to the best of our knowledge. DFT studies indicate that the high ORR activities on the PtCo NWs mainly originate from the hollow sites on the [110] and [310] high-index facets of NWs. Most significantly, without sacrificing the ORR activities, they show good stability for ORR over the long-term course of 20,000 cycles. Therefore, our finding may open up opportunities for the rational design of practically relevant catalysts with excellent activity and superior durability, which could impact broad areas such as fine chemical productions, fuel cells, batteries and beyond.

## Methods

### Preparation of hierarchical Pt_3_Co NWs

In a typical preparation of hierarchical Pt_3_Co NWs, platinum(II) acetylacetonate (Pt(acac)_2_, 10 mg), cobalt (III) acetylacetonate (Co(acac)_3_, 9.0 mg), CTAC (32.0 mg), glucose (60.0 mg) and 5 ml oleylamine were added into a vial (volume: 35 ml). After the vial had been capped, the mixture was ultrasonicated for 90 min. The resulting homogeneous dark green mixture was then heated from room temperature to 160 °C in 30 min and maintained at 160 °C for 8 h in an oil bath, before it was cooled to room temperature. The resulting colloidal products were collected by centrifugation and washed three times with an ethanol/cyclohexane mixture. The synthesis conditions for hierarchical Pt_7_Co and Pt_10_Co NWs were similar to that of hierarchical Pt_3_Co NWs except changing the amount of Co(acac)_3_ to 3.0 and 1.5 mg, respectively.

### Characterization

PXRD patterns were collected using an X'Pert-Pro X-ray powder diffractometer equipped with a Cu radiation source (*λ*=0.15406, nm). The morphology and size of the NWs were determined by TEM (Hitachi, HT7700) at 120 kV. Selected-area electron diffraction, HRTEM, HAADF-STEM and the 3D visualization of tomographic reconstruction tests were conducted on a JEOL-2100F transmission electron microscope at an acceleration voltage of 200 kV. High-resolution STEM imaging and elemental mapping analysis were carried out on a Hitachi HD2700C (200 kV) STEM with a probe aberration corrector, at the Center for Functional Nanomaterials, Brookhaven National Lab. Low-resolution energy dispersive X-ray spectroscopy was performed on a scanning electron microscope (Hitachi, S-4700). All the XPS spectra of the hierarchical Pt-Co NWs were collected by XPS (Thermo Scientific, ESCALAB 250 XI). The concentration of all the catalysts was determined by the ICP-AES (710-ES, Varian). The catalysts after the stability tests were collected and scratched off the electrode with the aid of sonication in ethanol for further TEM characterization.

### Electrochemical measurements

The catalysts were redispersed in a mixture solvent containing isopropanol and Nafion (5%; v-v=1:0.005) to form a homogeneous catalyst ink by sonicating for 30 min. The concentration of Pt was fixed to be 0.25 mg ml^−1^ based on ICP-AES measurement. Five microlitres of the dispersion was transferred onto the GC electrode. The Pt/C catalyst (20 wt%, 2–5 nm Pt NPs on Vulcan XC-72 carbon support, JM), was prepared by sonicating its ink in an aqueous dispersion (1.25 mg ml^−1^). Five microlitres of the dispersion was transferred onto the GC electrode with the loading amount of metal at 1.25 μg.

A CHI660E electrochemical analyzer (CHI Instruments) was used for testing the MOR and EOR of different catalysts. The MOR and EOR measurements were carried out in 0.1 M HClO_4_+0.2 M methanol and 0.1 M HClO_4_+0.2 M ethanol at room temperature, respectively. The CV technique and conventional three-electrode system were applied to evaluate the electrochemical properties of various catalysts. A leak-free saturated calomel electrode was used as the reference electrode and a Pt wire was used as the counter electrode. The ECSA was determined by integrating the hydrogen adsorption charge on the CVs at the rate of 50 mV s^−1^ at room temperature in N_2_-saturated 0.1 M HClO_4_ solution. In the CV measurements for MOR and EOR, the electrode potential was scanned at a rate of 50 mV s^−1^. All the catalyst electrodes were cleaned before data collection with a steady-state CV. The current was normalized to Pt amount and ECSA to get mass and specific activities, respectively.

The electrochemical measurements for ORR were performed using a glassy-carbon Rotating Disk Electrode (Pine Research Instrumentation, diameter: 5 mm, area: 0.196 cm^2^) connected to an installation of rotating electrode speed control (Pine Research Instrumentation, model: AFMSRCE). A leak-free saturated calomel electrode was used as the reference electrode and a Pt wire was used as the counter electrode. The electrolyte was 0.1 M HClO_4_. The loading amount of Pt for the hierarchical Pt_10_Co NWs/C, Pt_7_Co NWs/C, Pt_3_Co NWs/C, Pt_3_Co NPs/C and the commercial Pt/C (JM) catalysts was all kept at 6.4 μg cm^−2^. ORR measurements were conducted in 0.1 M HClO_4_ solutions purged with the saturated O_2_ during the measurements. The scan and rotation rates for ORR measurements were 10 mV s^−1^ and 1,600 r.p.m., respectively. In the ORR polarization curves, the current densities were normalized in reference to the geometric area of the glassy carbon electrode (0.196 cm^2^). For each catalyst, the kinetic current was normalized to the loading amount of Pt and ECSA in order to generate mass and specific activities, respectively. The accelerated durability tests were performed at room temperature in 0.1 M HClO_4_ solutions by applying the cyclic potential sweeps between 0.6 and 1.1 V versus RHE at a sweep rate of 100 mV s^−1^ for 20,000 cycles.

### DFT calculations

The oxygen adsorption energy *E*_O_ was defined as *E*_O_=*E*[surf+O]−*E*[surf]−*E*[O_2_]/2, where *E*[surf +O] and *E*[surf] are the total energies of the [110] or [310] surface with and without the O adsorbate, respectively. *E*[O_2_] is the total energy of an oxygen molecule. A four-layer slab was used in the calculations. The atoms in the top two layers were fully relaxed while the rest of the atoms were fixed in their equilibrium positions. The DFT calculations were carried out using the VASP package^44^,^45^ with the projector augmented wave pseudopotentials^46^ and Perdew–Burke–Ernzerh of generalized gradient approximation.^47^ An energy cutoff of 400 eV was used for the plane-wave basis set. The Brillouin zone was sampled on the basis of the Monkhorst–Pack scheme^48^ with a 7 × 5 × 1 k-point mesh. The force convergence criterion for atomic relaxation was 0.02 eV Å^−1^.

### Data availability

All relevant data are available from the authors on request.

## Additional information

**How to cite this article:** Bu, L. *et al*. Surface engineering of hierarchical platinum-cobalt nanowires for efficient electrocatalysis. *Nat. Commun.* 7:11850 doi: 10.1038/ncomms11850 (2016).

## Supplementary Material

Supplementary Information Supplementary Figures 1-26 and Supplementary Tables 1-3.
