# Supplementary material for: Surface engineering of hierarchical platinum-cobalt nanowires for efficient electrocatalysis
Source: Nat Commun. 2016 Jun 29;7:11850. doi: 10.1038/ncomms11850 (PMC4931244; doi:10.1038/ncomms11850)
Supplement: Supplementary Information — Supplementary Figures 1-26 and Supplementary Tables 1-3. [file ncomms11850-s1.pdf]

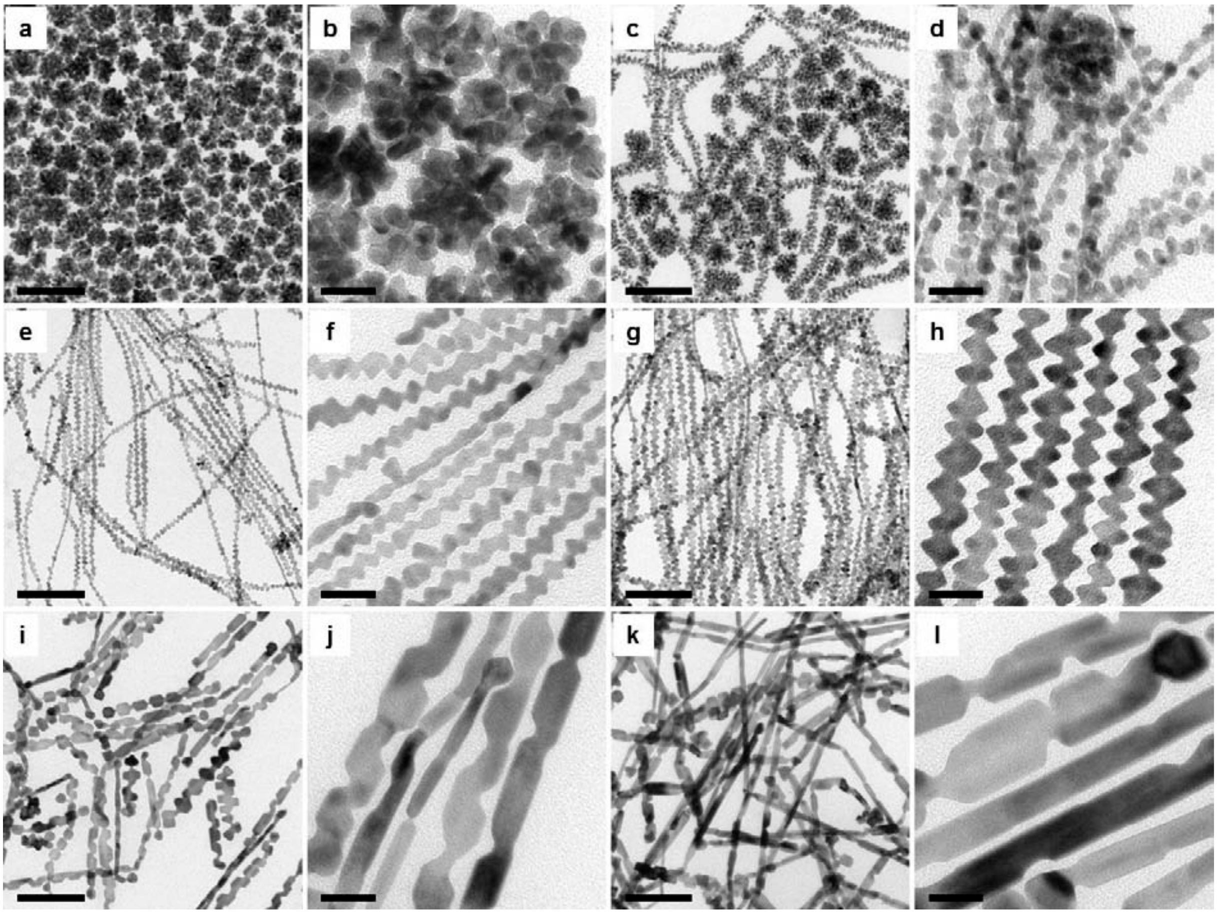

Supplementary Figure 1. Representative TEM images of the products with the same reaction conditions as that of hierarchical Pt_3Co NWs except the use of (a, b) 0 mg CTAC, (c, d) 8 mg CTAC, (e, f) 16 mg CTAC, (g, h) 32 mg CTAC, (i, j) 64 mg CTAC and (k, l) 128 mg CTAC. The scale bars in (a), (c), (e), (g), (i) and (k) are 100 nm. The scale bars in (b), (d), (f), (h), (j) and (l) are 20 nm.

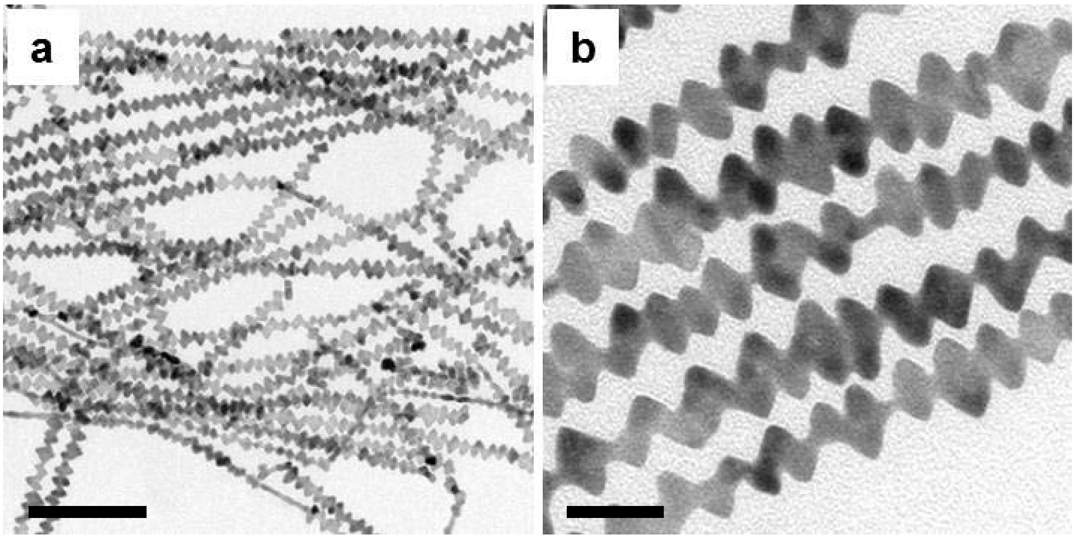

Supplementary Figure 2. Representative TEM images of the products with the same reaction conditions as that of hierarchical Pt_3Co NWs except changing CTAC with CTAB. The scale bar in (a) is 100 nm. The scale bar in (b) is 20 nm.

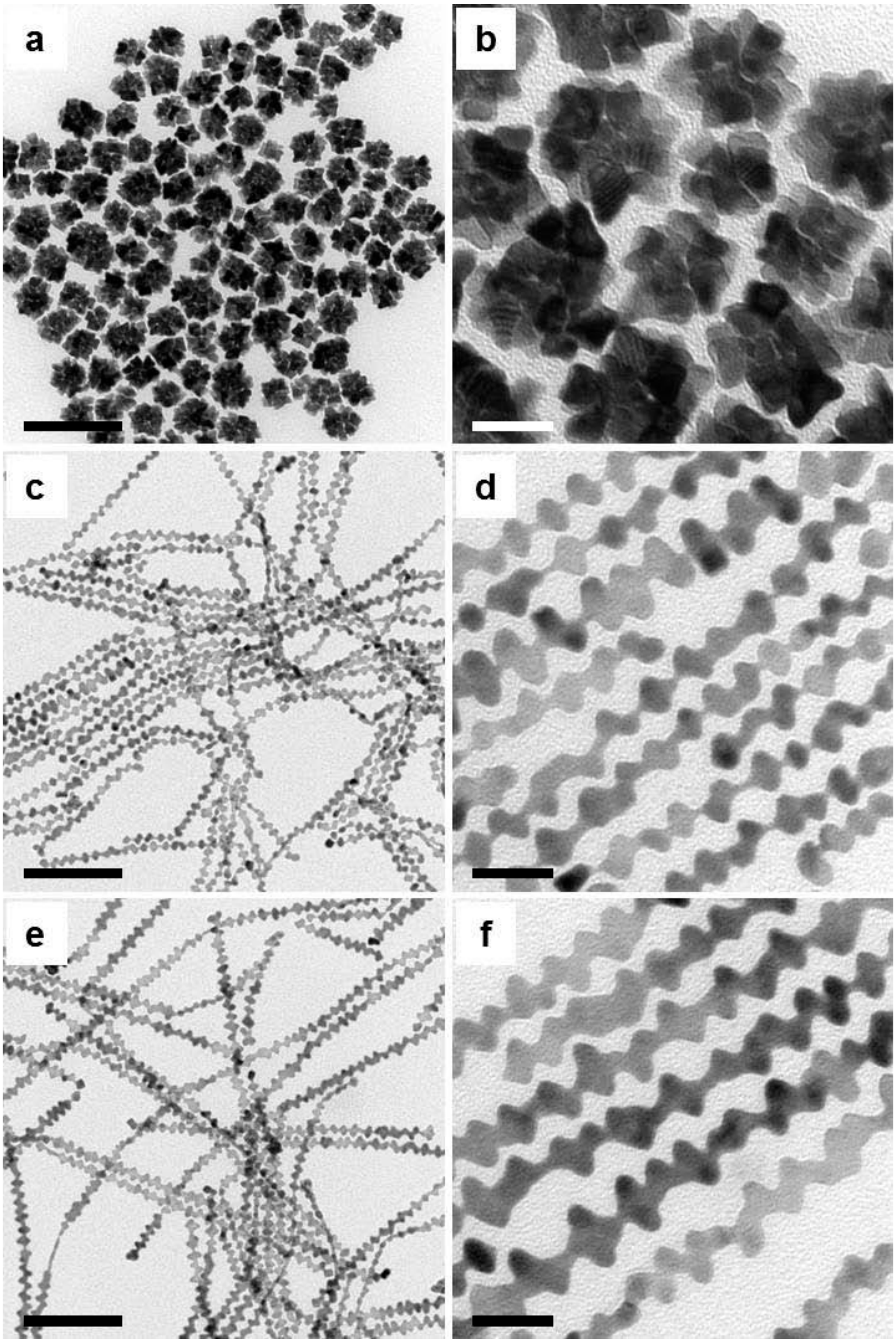

Supplementary Figure 3. Representative TEM images of the products with the same reaction conditions as that of hierarchical Pt_3Co NWs except the use of (a, b) 0 mg glucose, (c, d) 30 mg glucose and (e, f) 90 mg glucose. The scale bars in (a), (c) and (e) are 100 nm. The scale bars in (b), (d) and (f) are 20 nm.

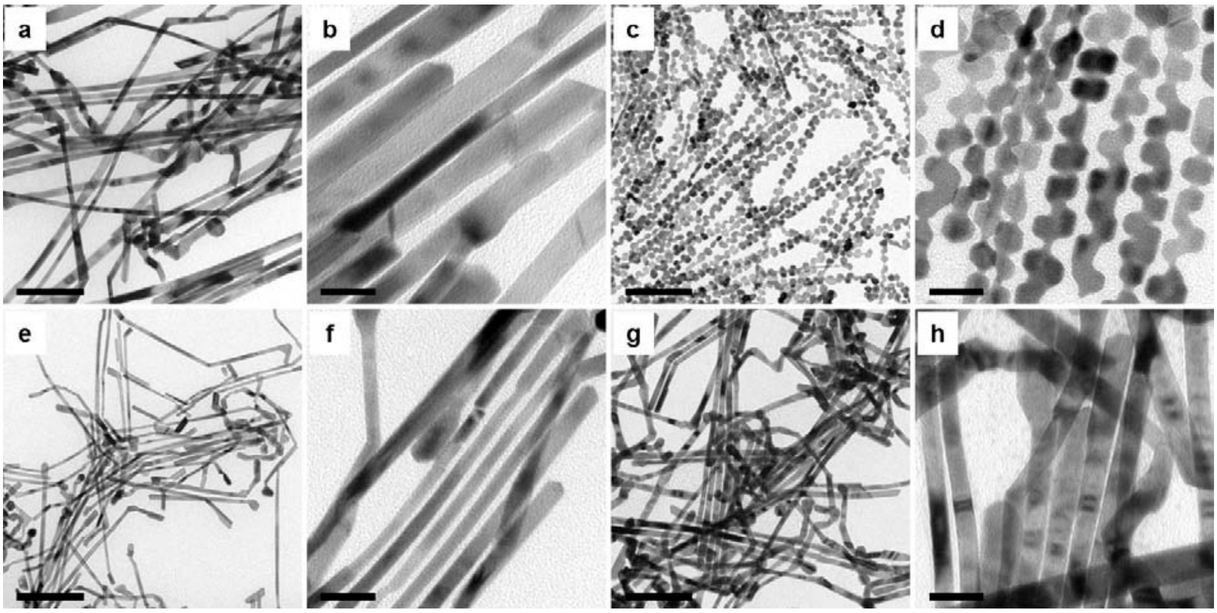

Supplementary Figure 4. Representative TEM images of the products with the same reaction conditions as that of hierarchical Pt_3Co NWs except the use of (a, b) 0 mg $Co(acac)_3$, (c, d) 12 mg $Co(acac)_3$, (e, f) 6.1 mg $Ca(acac)_2$ and (g, h) 3.5 mg Na(acac). The scale bars in (a), (c), (e) and (g) are 100 nm. The scale bars in (b), (d), (f) and (h) are 20 nm.

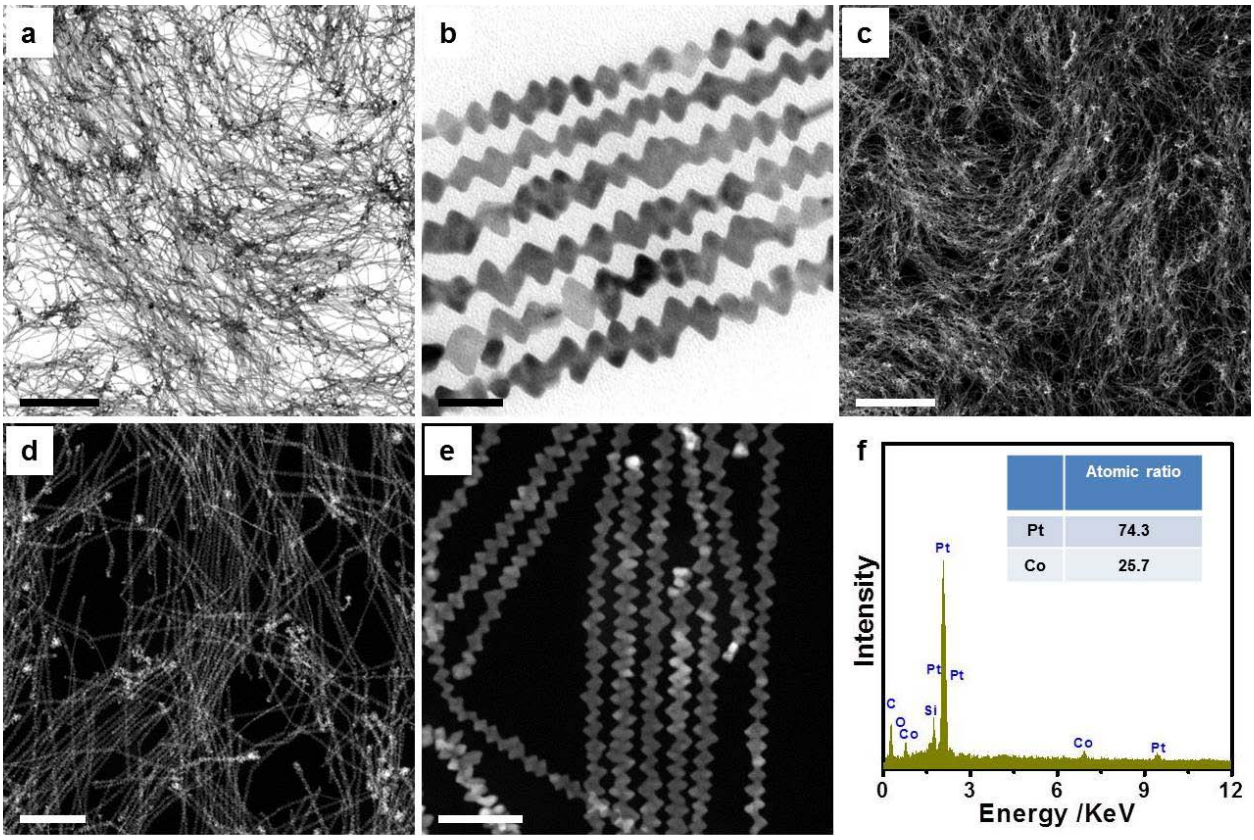

Supplementary Figure 5. Additional (a-b) TEM, (c-e) STEM images, and (f) SEM-EDX image of the hierarchical Pt_3Co NWs. The scale bars in (a), (c), (e) and (g) are 100 nm. The scale bars in (a), (b), (c), (d) and (e) are 1.0 μm, 20 nm, 2.0 μm, 200 nm and 50 nm, respectively.

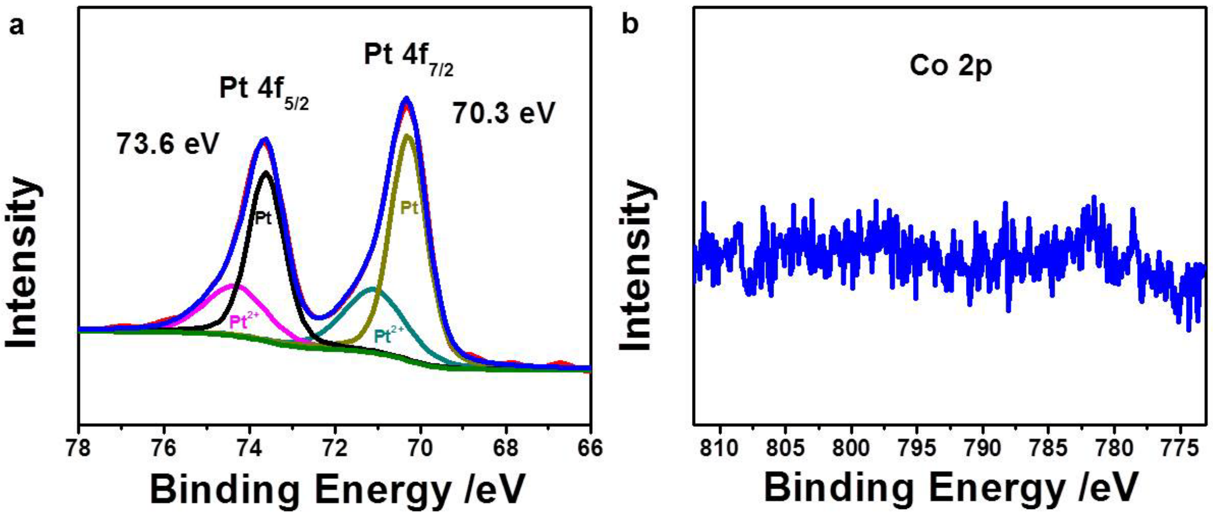

Supplementary Figure 6. (a) Pt 4f and (b) Co 2p XPS spectra of hierarchical Pt_3Co NWs/C. The molar ratio was around 95.2/4.8, as determined by XPS.

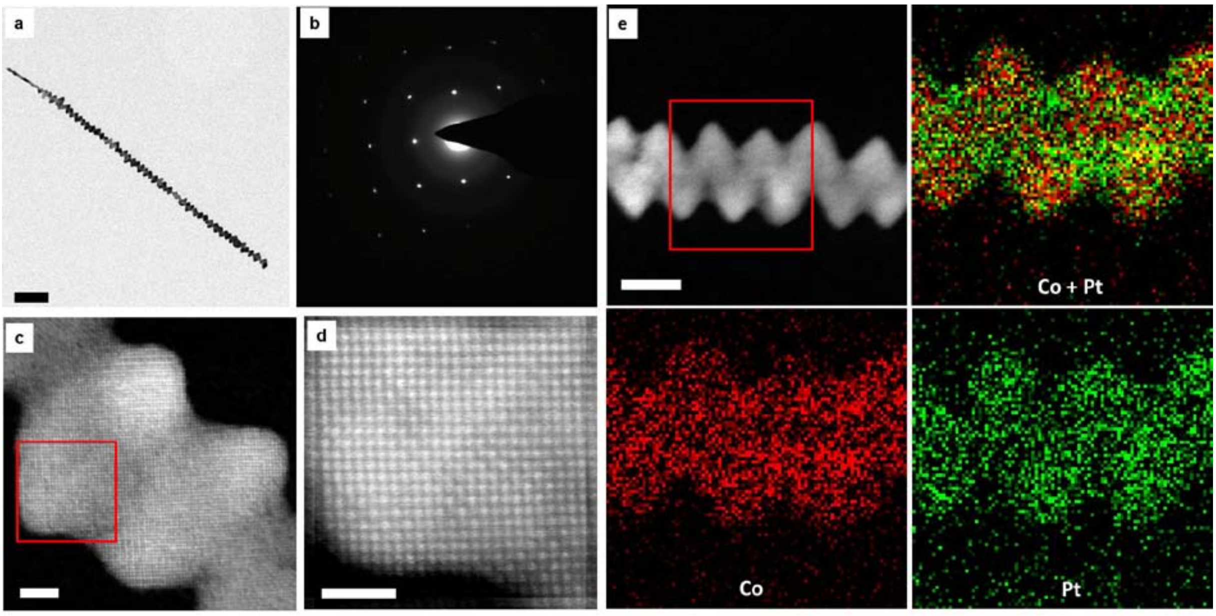

Supplementary Figure 7. (a) TEM image and (b) corresponding SAED of an individual hierarchical Pt_3Co NWs. (c) HR-STEM image, (d) enlarged HR-STEM image and (e) HAADF-STEM image and elemental mappings of hierarchical Pt_3Co NWs. The scale bars in (a), (c), (d) and (e) are 50 nm, 2 nm, 2 nm, and 10 nm, respectively.

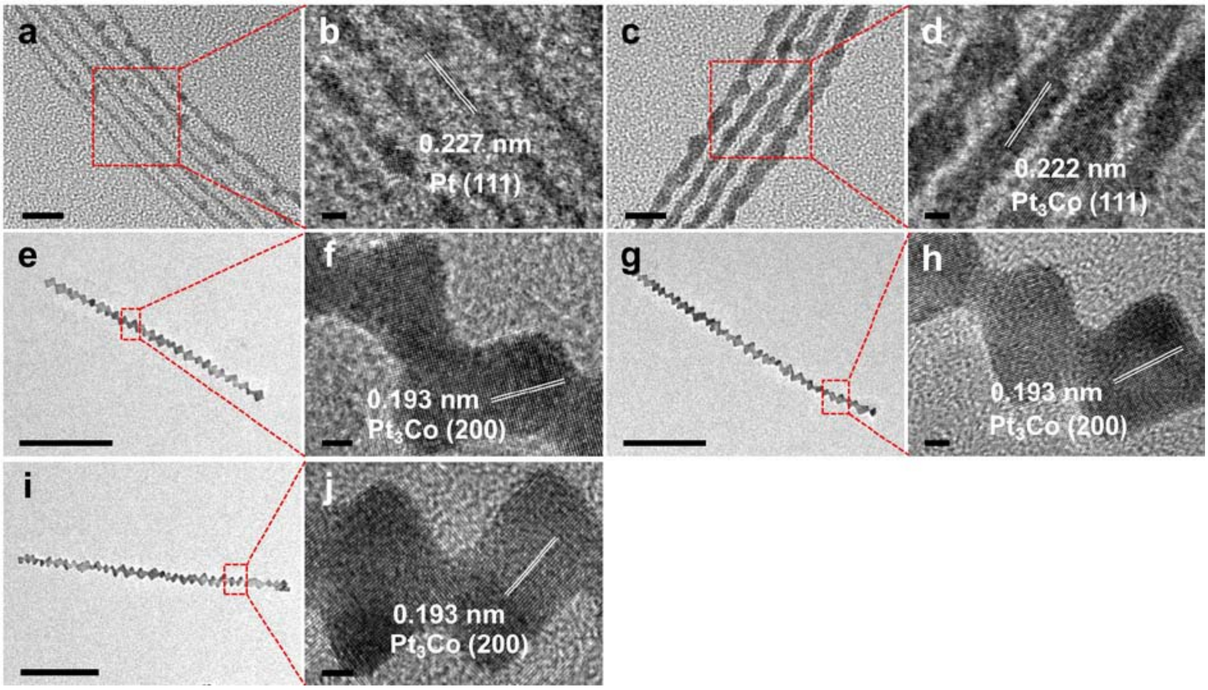

Supplementary Figure 8. Representative TEM images and corresponding HRTEM images of the hierarchical Pt_3Co NWs intermediates collected from (a) 0.5 h, (b) 1 h, (c) 3 h, (d) 5 h, and (e) 8 h. The scale bars in (a) and (c) are 10 nm. The scale bars in (e), (g) and (i) are 100 nm. The scale bars in (b), (d), (f), (h) and (j) are 2 nm.

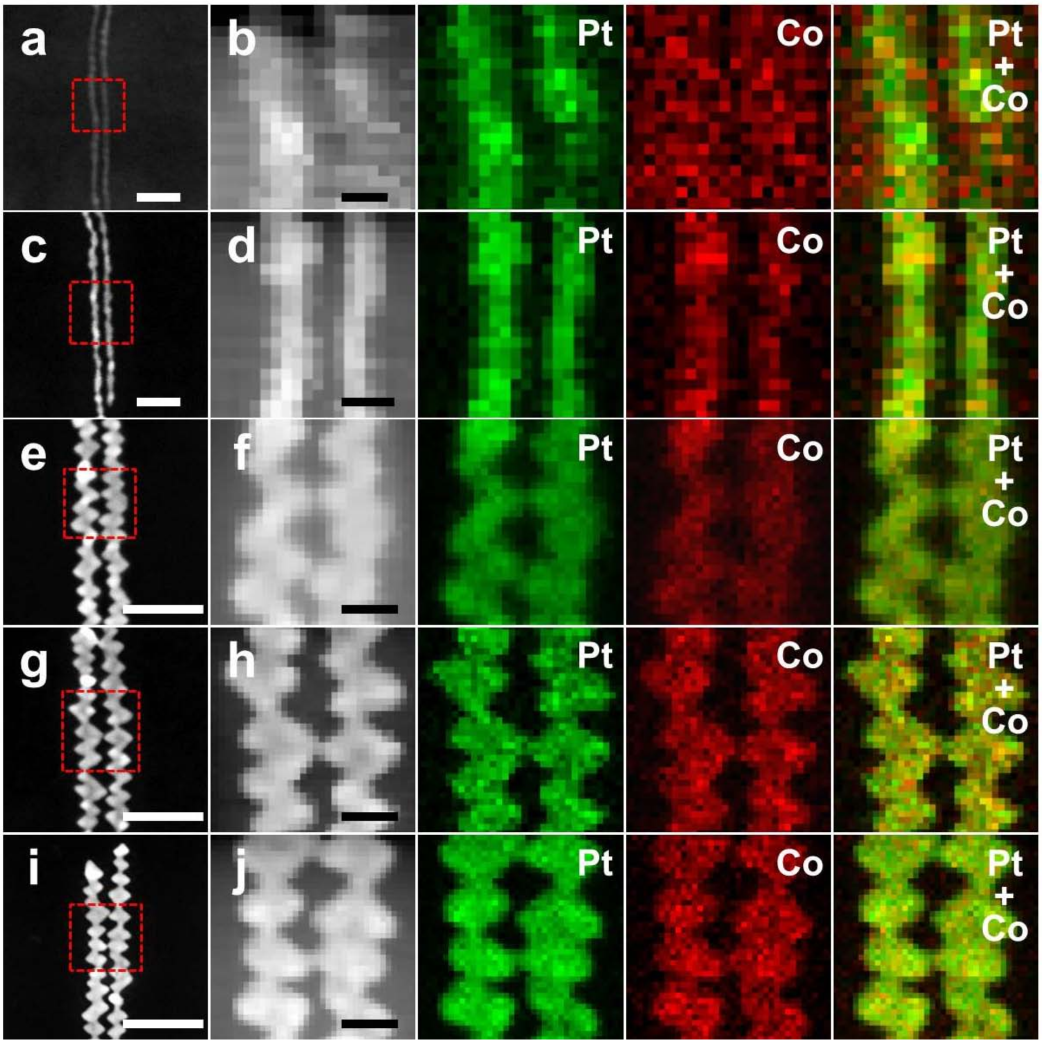

Supplementary Figure 9. Representative HAADF-STEM images and elemental mappings of the hierarchical Pt_3Co NWs intermediates collected from (a, b) 0.5 h, (c, d) 1 h, (e, f) 3 h, (g, h) 5 h, and (i, j) 8 h. The scale bars in (a) and (c) are 20 nm. The scale bars in (b) and (d) are 2 nm and 5 nm, respectively. The scale bars in (e), (g) and (i) are 50 nm. The scale bars in (f), (h) and (j) are 10 nm.

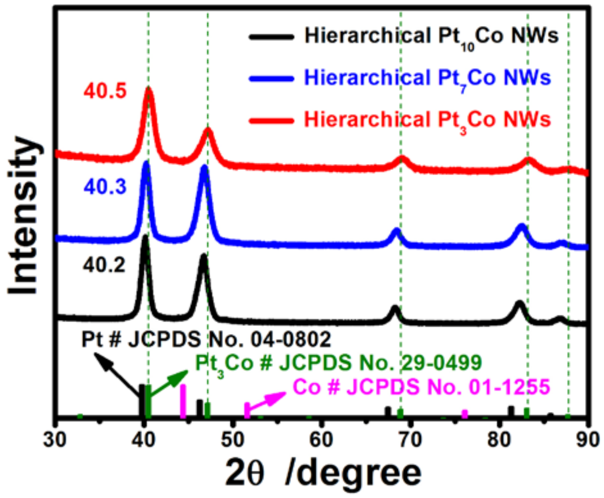

Supplementary Figure 10. XRD patterns of three kinds of hierarchical Pt-Co NWs.

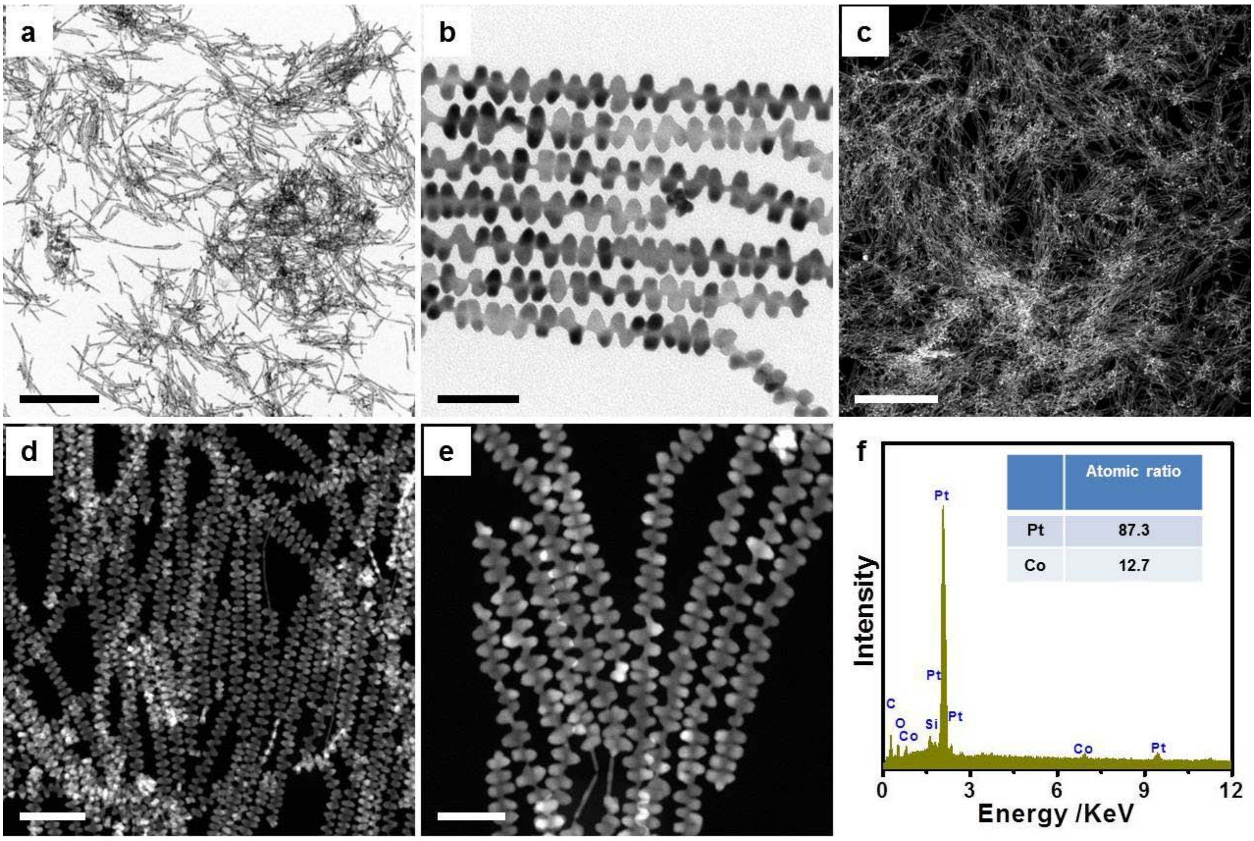

Supplementary Figure 11. Additional (a-b) TEM, (c-e) STEM and (f) SEM-EDX images of the hierarchical Pt_7Co NWs. The scale bars in (a), (b), (c), (d) and (e) are 1.0 μm, 50 nm, 1.0 μm, 100 nm and 50 nm, respectively.

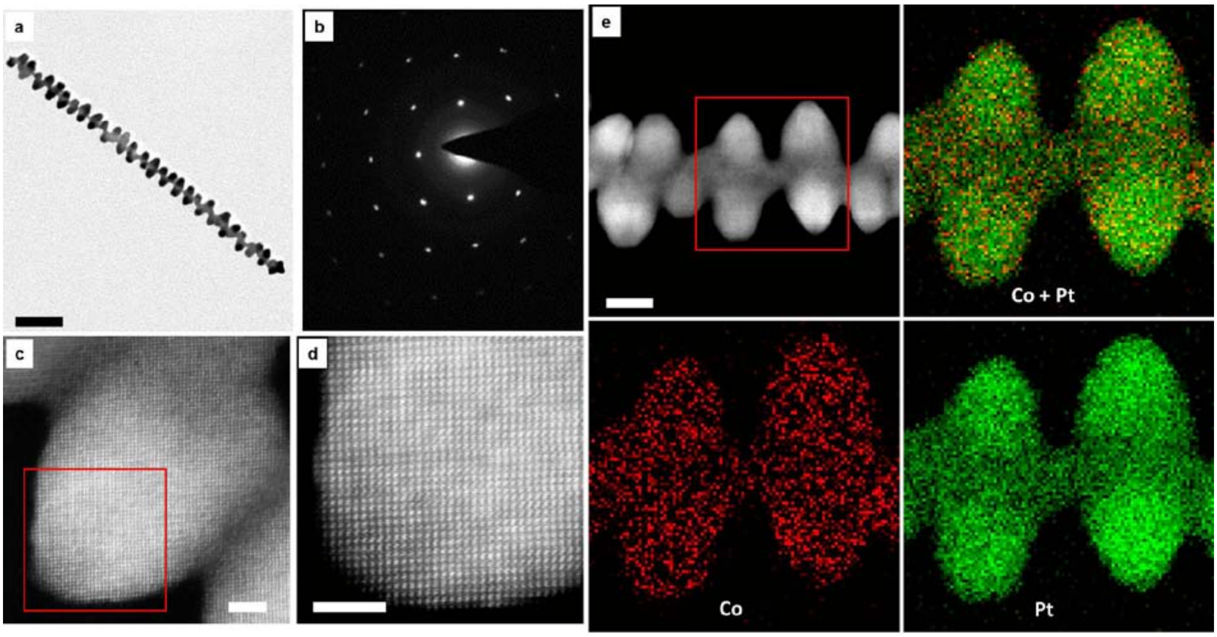

Supplementary Figure 12. (a)TEM image and (b) corresponding SAED of an individual hierarchical Pt_7Co NWs. (c) HR-STEM image, (d) enlarged HR-STEM image and (e) HAADF-STEM image and elemental mappings of the hierarchical Pt_7Co NWs. The scale bars in (a), (c), (d) and (e) are 50 nm, 2 nm, 2 nm and 10 nm, respectively.

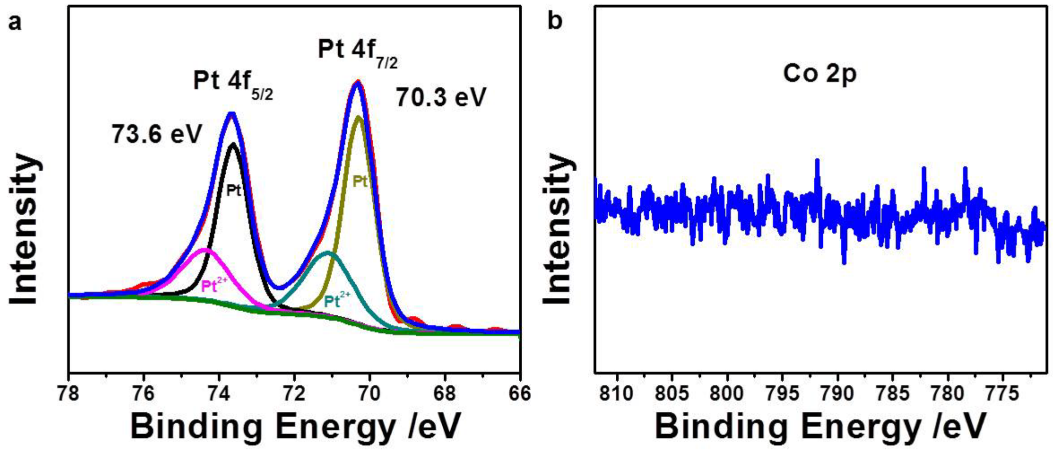

Supplementary Figure 13. (a) Pt 4f and (b) Co 2p XPS spectra of hierarchical Pt_7Co NWs/C. The molar ratio was around 96.9/3.1, as determined by XPS.

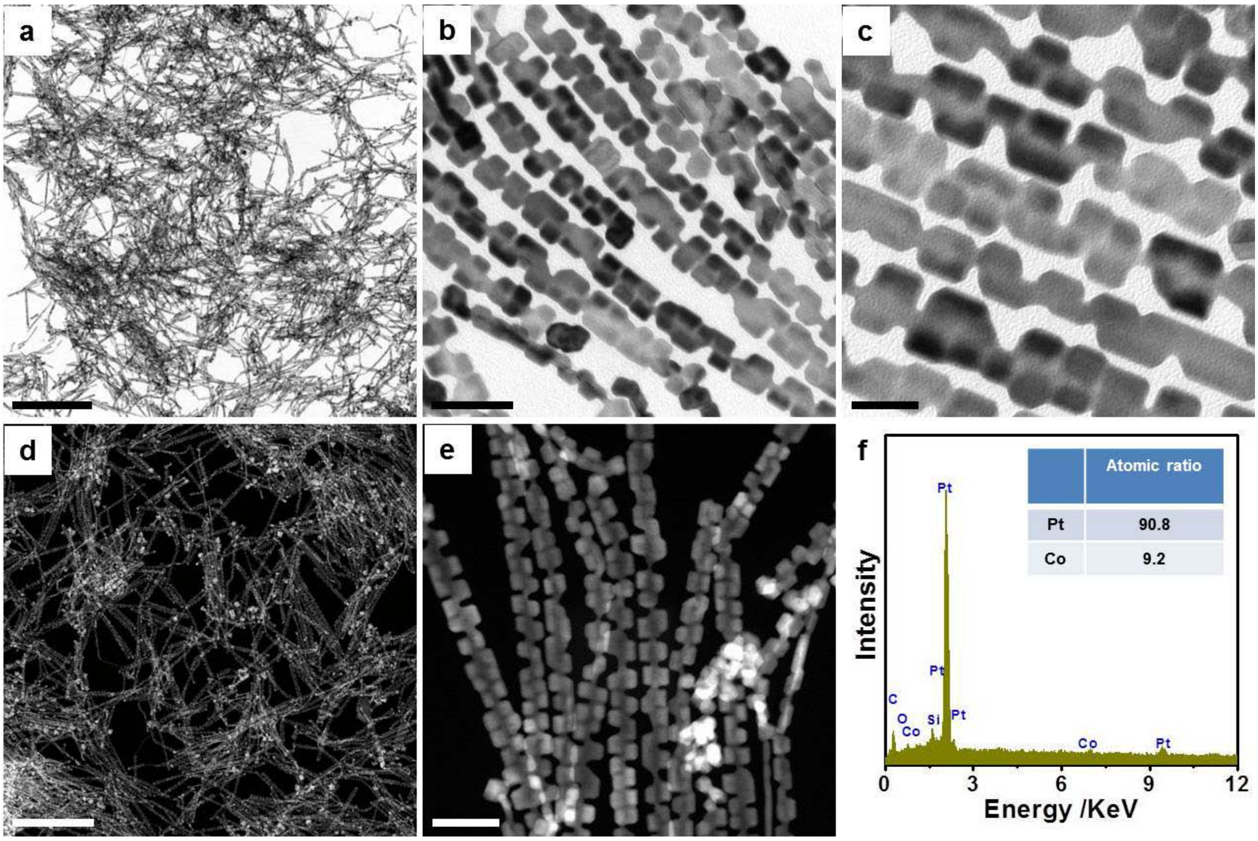

Supplementary Figure 14. Additional (a-c) TEM, (d-e) STEM and (f) SEM-EDX image of the hierarchical $Pt_{10}Co$ NWs. The scale bars in (a), (b), (c), (d) and (e) are 1.0 μm, 50 nm, 20 nm, 500 nm and 50 nm, respectively.

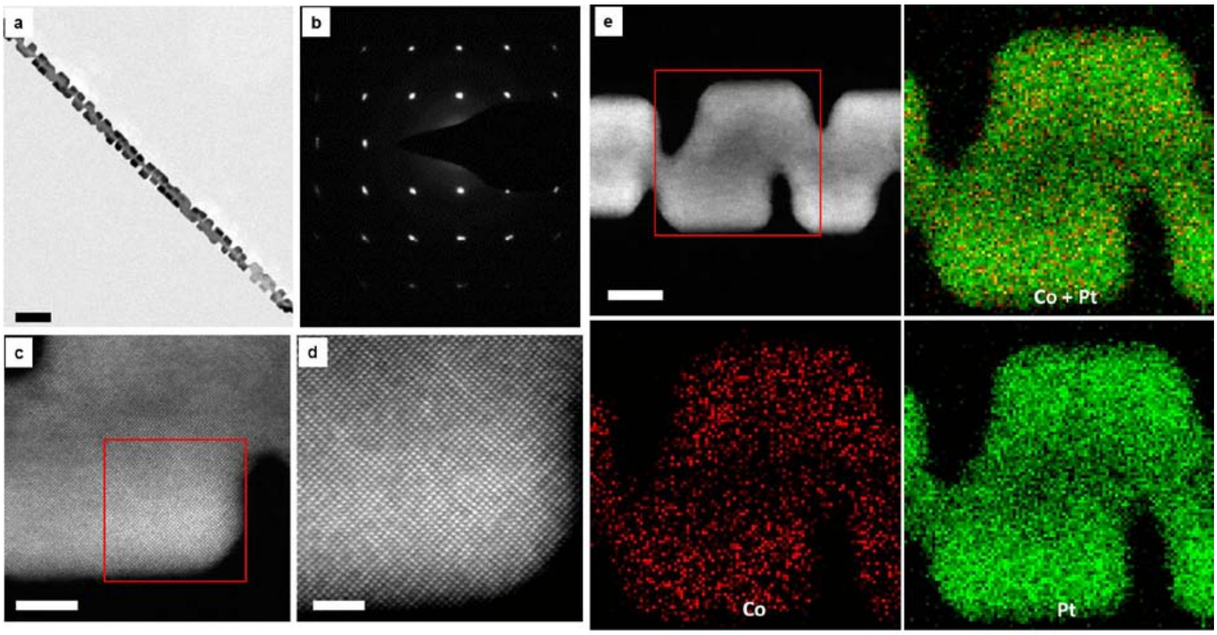

Supplementary Figure 15. (a)TEM image and (b) corresponding SAED of an individual hierarchical $Pt_{10}Co$ NWs. (c) HR-STEM image, (d) enlarged HR-STEM image and (e) HAADF-STEM image and elemental mappings of the hierarchical $Pt_{10}Co$ NWs. The scale bars in (a), (c), (d) and (e) are 50 nm, 5 nm, 2 nm and 10 nm, respectively.

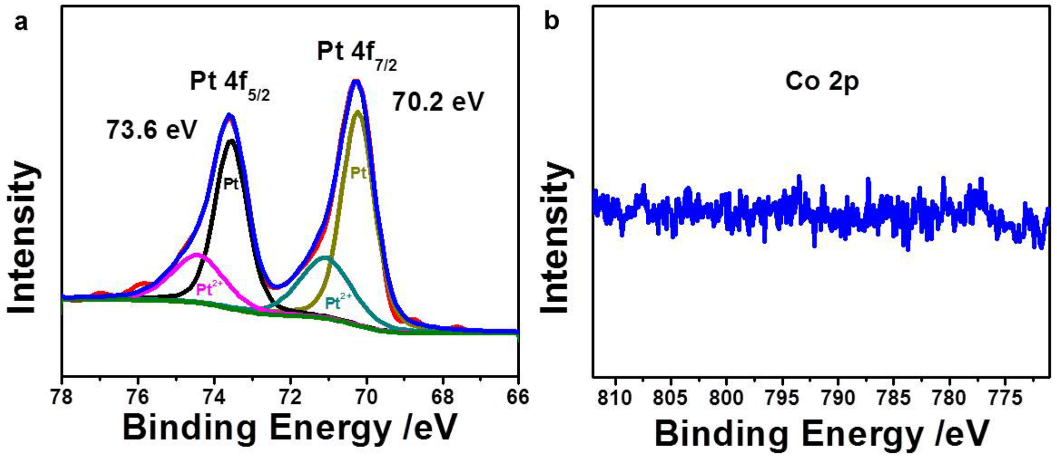

Supplementary Figure 16. (a) Pt 4f and (b) Co 2p XPS spectra of hierarchical $Pt_{10}Co$ NWs/C. The molar ratio was 98.5/1.5, as determined by XPS.

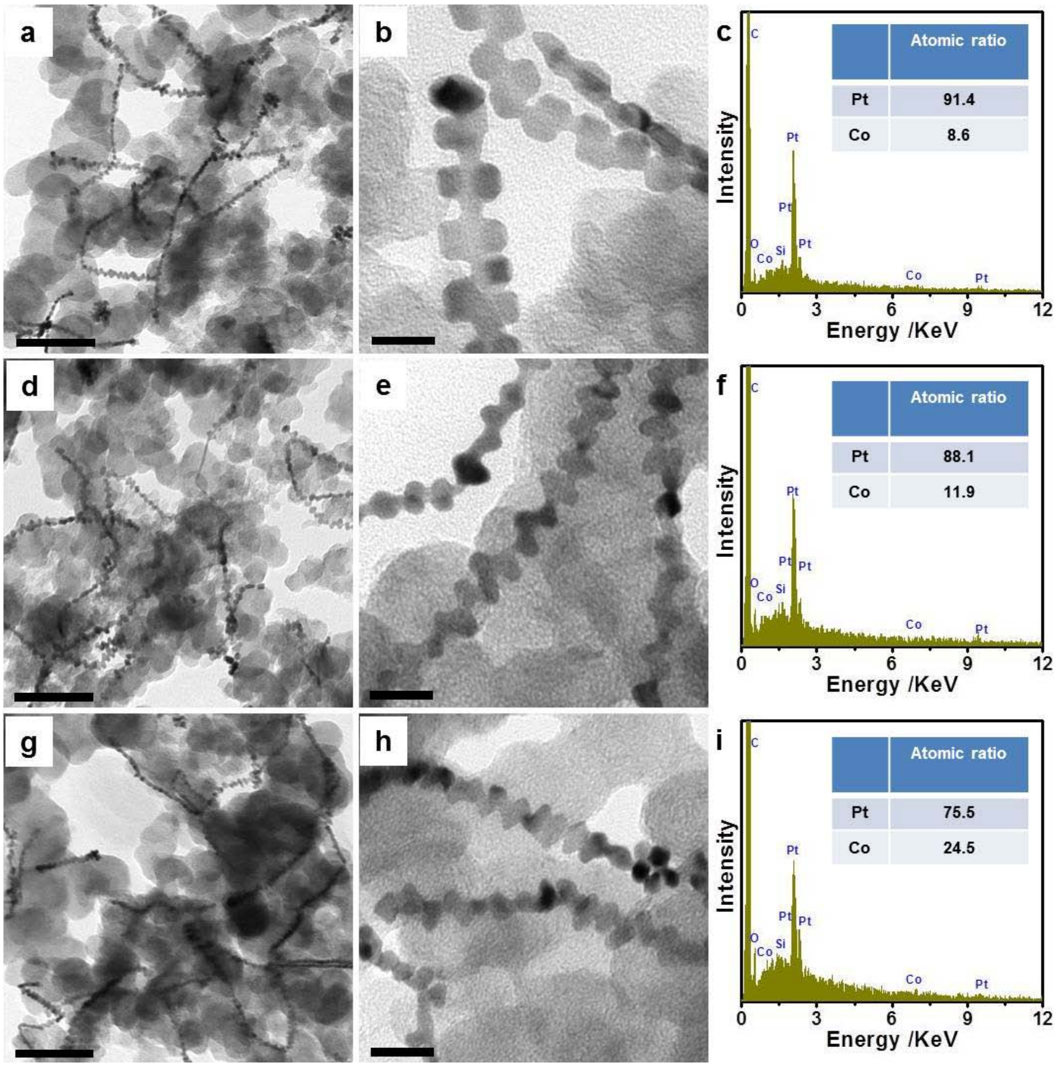

Supplementary Figure 17. Representative TEM images and SEM-EDX of the hierarchical $Pt_{10}Co/C$ catalyst (a-c), Pt_7Co/C catalyst (d-f) and Pt_3Co/C catalyst (g-i). The scale bars in (a), (d) and (g) are 100 nm. The scale bars in (b), (e) and (h) are 20 nm.

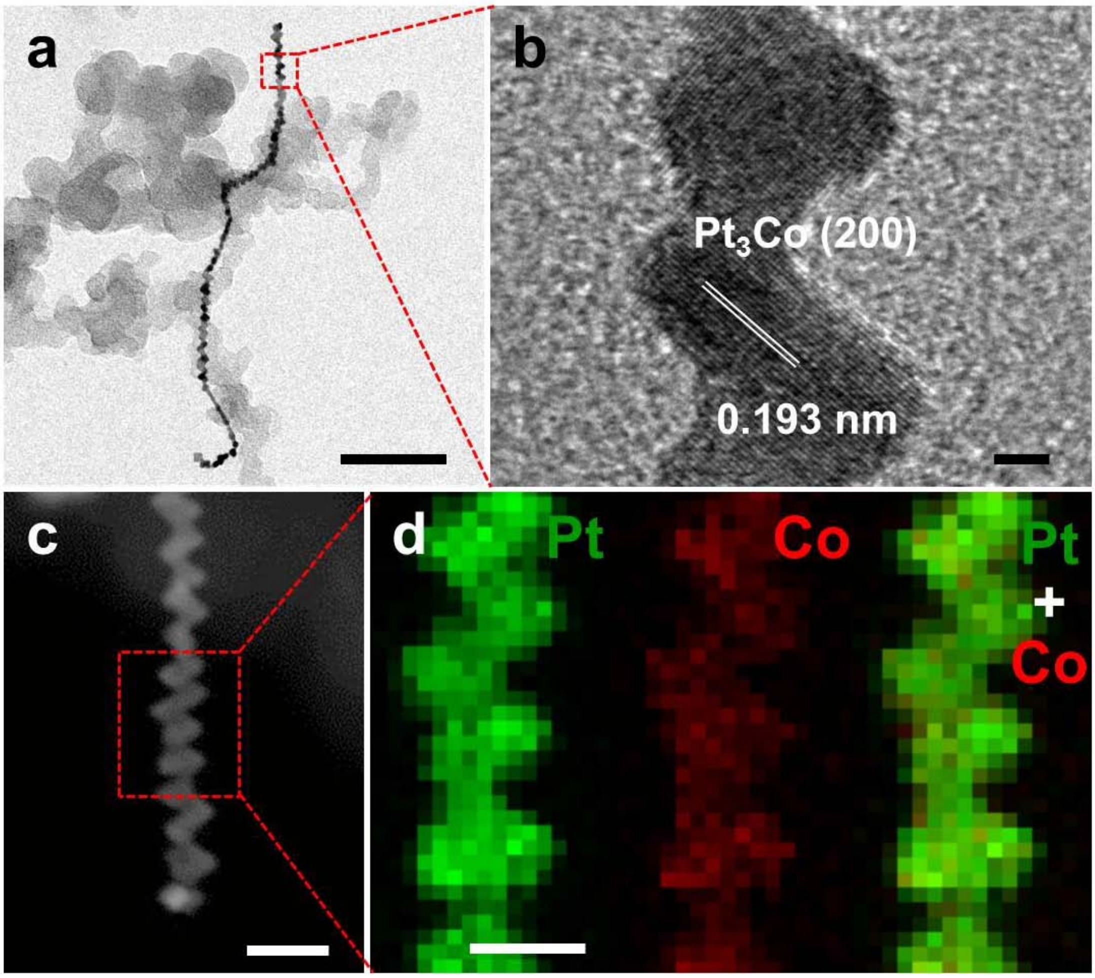

Supplementary Figure 18. Representative (a) TEM image, (b) HRTEM, (c) STEM image, and (d) elemental mappings of the hierarchical Pt_3Co/C catalyst. The scale bars in (a), (b), (c) and (d) are 100 nm, 2 nm, 20 nm and 10 nm, respectively.

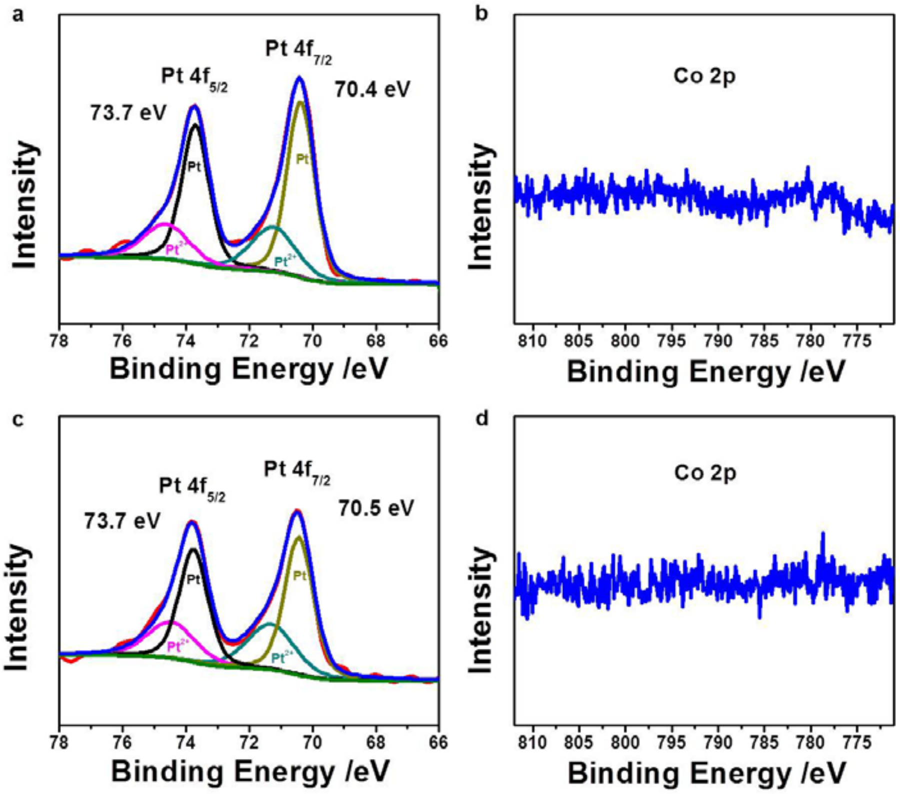

Supplementary Figure 19. (a, c) Pt 4f and (b, d) Co 2p XPS spectra of hierarchical Pt_3Co NWs/C before (a, b) and after (c, d) 20000 potential cycles between 0.6 and 1.1 V *vs.* RHE. The molar ratio was largely maintained (from 95.4/4.6 to 96.5/3.5).

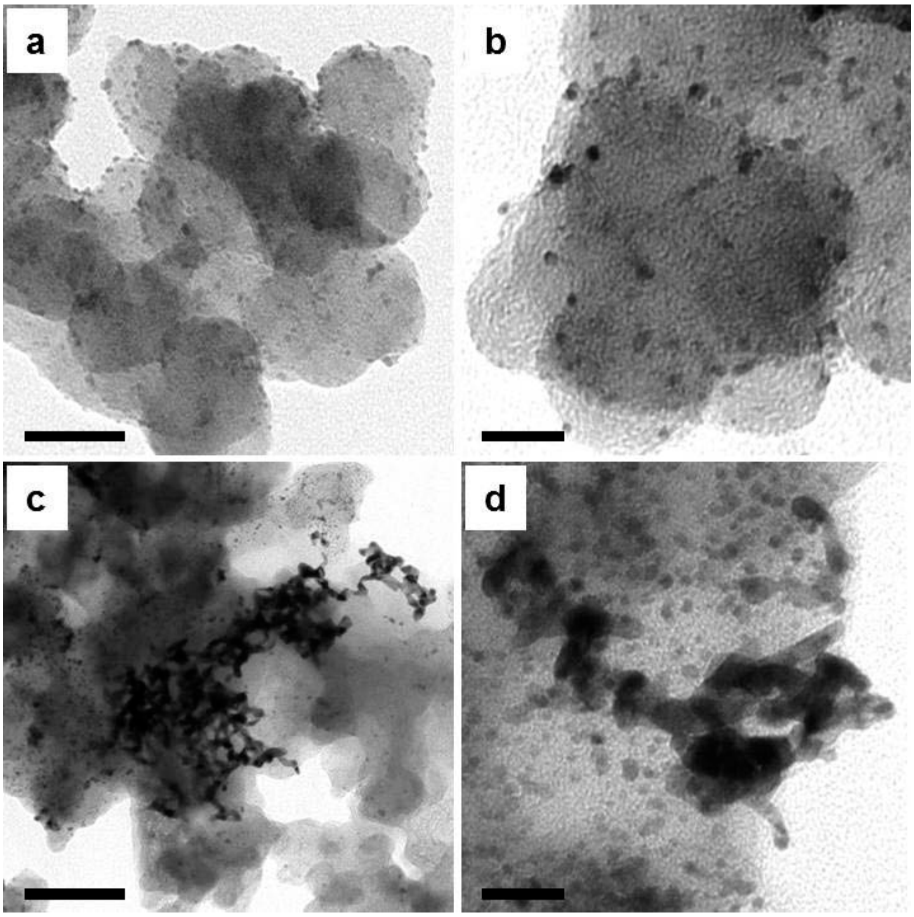

Supplementary Figure 20. Representative TEM images of Pt/C catalyst (a, b) before and after (c, d) 20000 potential cycles between 0.6 and 1.1 V *vs.* RHE. The scale bars in (a) and (c) are 100 nm. The scale bars in (b) and (d) are 20 nm.

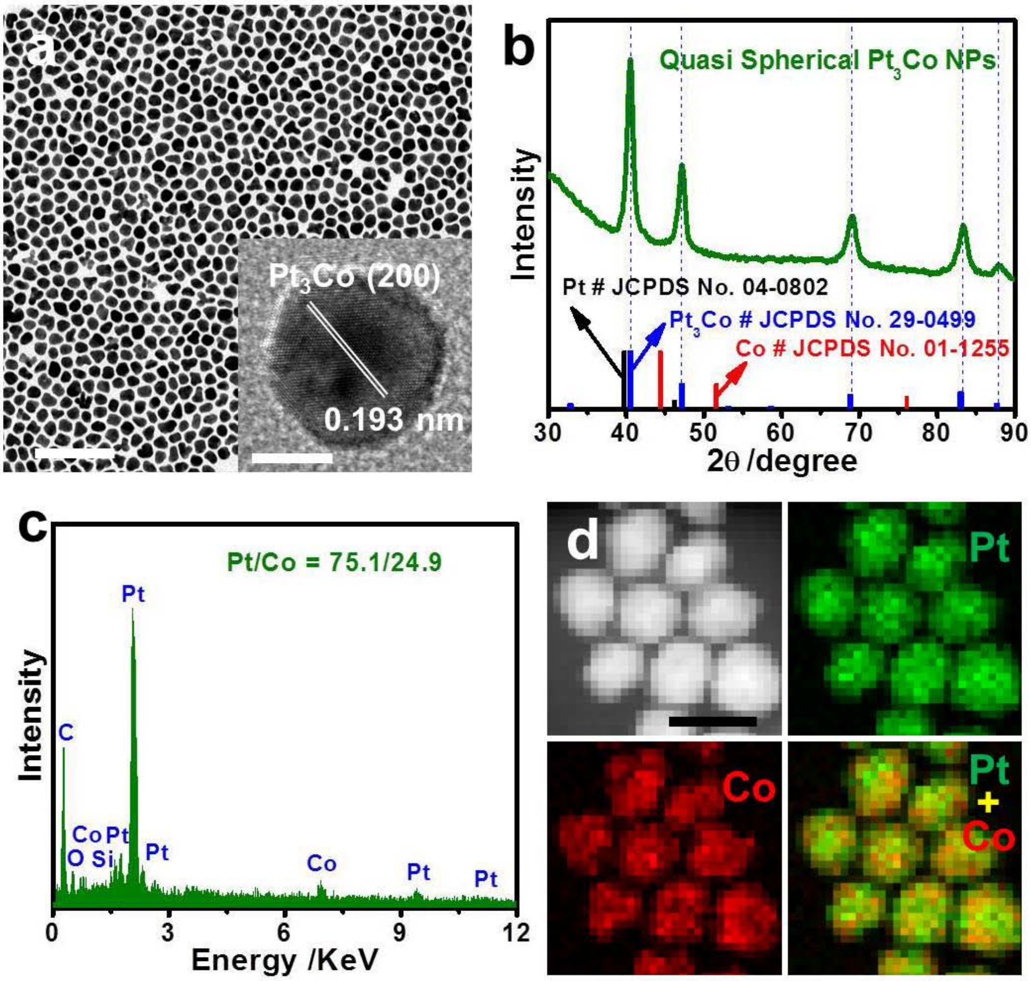

Supplementary Figure 21. Representative (a) TEM image, (b) PXRD pattern, (c) SEM-EDX, and (d) HAADF-STEM elemental mappings of the home-made Pt_3Co NPs. Inset in (a) shows the HRTEM image of an individual Pt_3Co NP. Synthesis of Pt_3Co NPs: platinum(II) acetylacetonate ($Pt(acac)_2$, 10 mg), cobalt (III) acetylacetonate ($Co(acac)_3$, 3.0 mg), ethanediol (EG, 0.1 mL), and 5 mL oleylamine were added into a vial (volume: 35 mL). After the vial had been capped, the mixture was ultrasonicated for 90 min. The resulting homogeneous mixture was then heated from room temperature to 190 °C in around 40 min and maintained at 190 °C for 8 h in an oil bath, before it was cooled to room temperature. The scale bars in (a), inset of (a) and (d) are 100 nm, 5 nm and 20 nm.

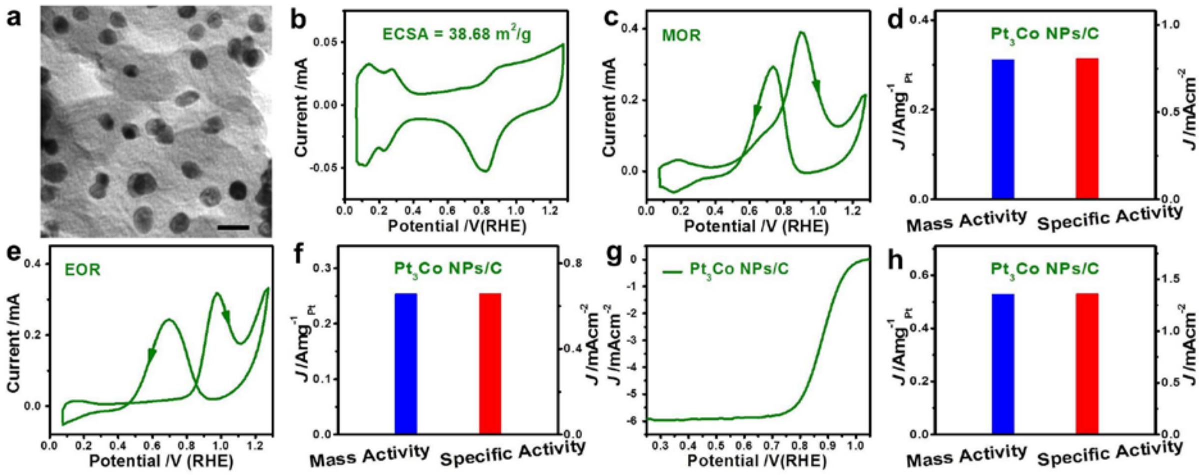

Supplementary Figure 22. (a) Representative TEM image and (b) CV of the Pt_3Co NPs/C catalyst in 0.1 M $HClO_4$ solution. (c) CV and (d) histogram of mass and specific activities of the as-obtained Pt_3Co NPs/C catalyst for MOR in 0.1 M $HClO_4$ + 0.2 M methanol solution. (e) CV and (f) histogram of mass and specific activities of the as-obtained Pt_3Co NPs/C catalyst for EOR in 0.1 M $HClO_4$ + 0.2 M ethanol solution. (g) ORR polarization curve and (h) ORR specific and mass activities of the as-obtained Pt_3Co NPs/C catalyst. The ORR polarization curve was recorded at room temperature in an O_2-saturated 0.1 M $HClO_4$ aqueous solution at a sweep rate of 10 mV s^{-1} and a rotation rate of 1600 rpm. The scale bar in (a) is 20 nm.

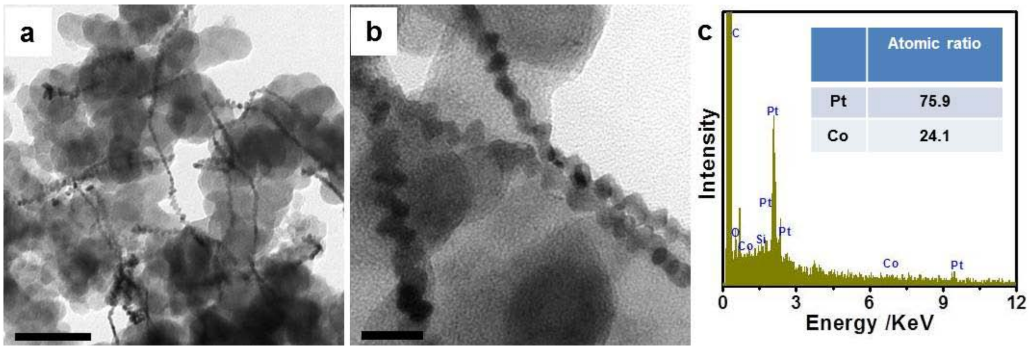

Supplementary Figure 23. Representative TEM images (a-b) and SEM-EDX (c) of the hierarchical Pt_3Co/C catalyst after 20000 potential cycles between 0.6 and 1.1 V *vs.* RHE. The scale bars in (a) and (b) are 100 nm and 20 nm, respectively.

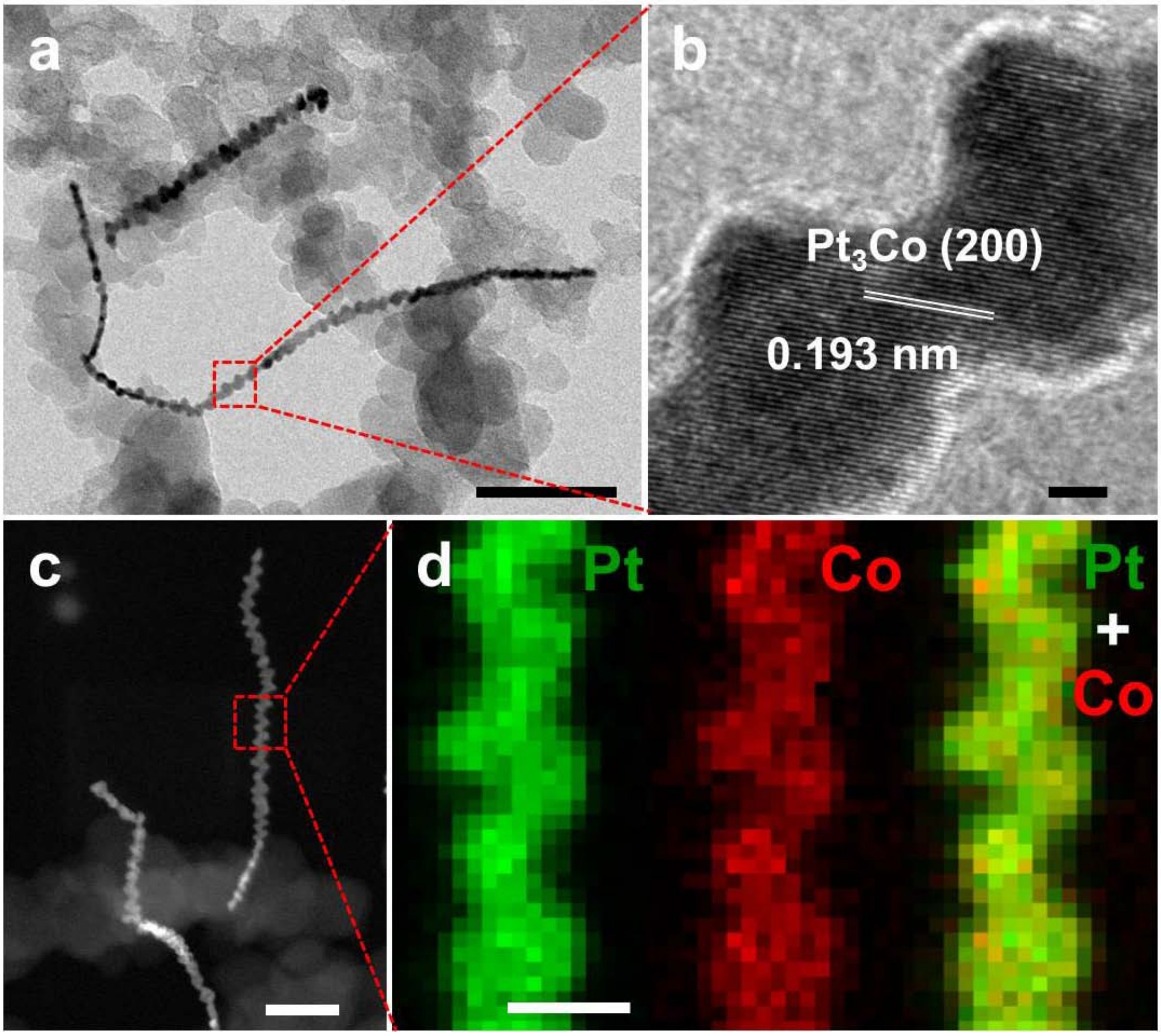

Supplementary Figure 24. Representative (a) TEM image, (b) HRTEM, (c) STEM image, and (d) elemental mappings of the hierarchical Pt_3Co/C catalyst after 20000 potential cycles between 0.6 and 1.1 V *vs.* RHE. The scale bars in (a), (b), (c) and (d) are 100 nm, 2 nm, 50 nm and 10 nm, respectively.

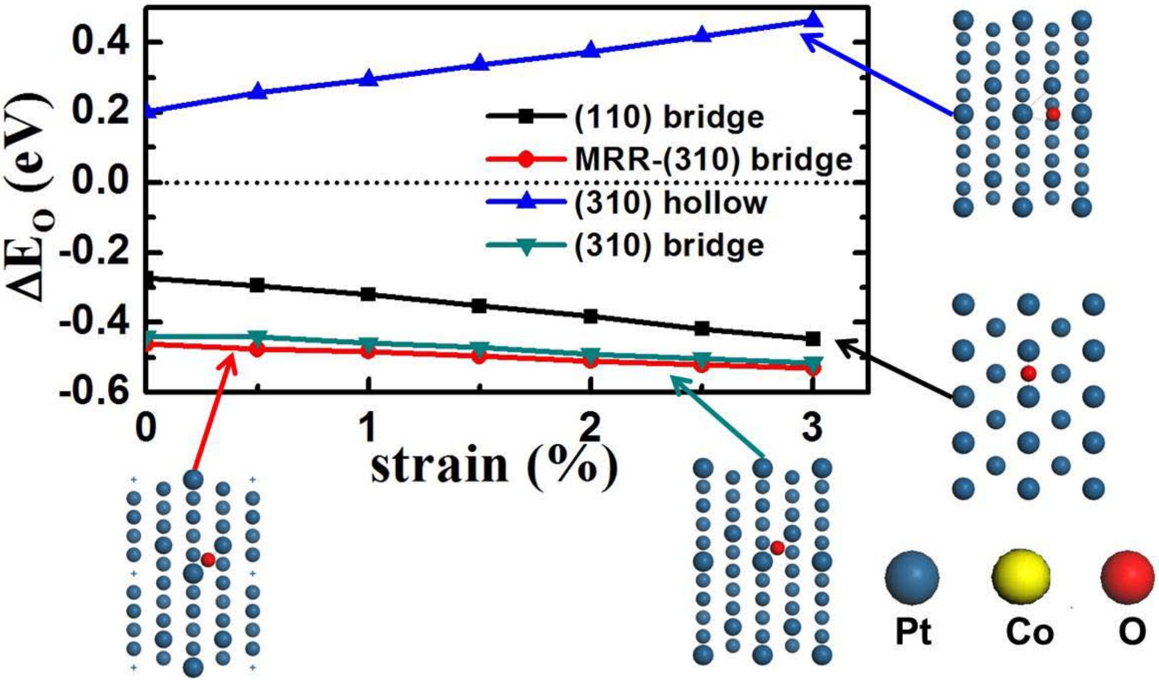

Supplementary Figure 25. Atomistic models of (110), (310) and (2×1) missing-row reconstructed (MRR) (310) surfaces and ΔE_O as a function of compressive strain. The horizontal dashed line indicates the optimalΔE_O value. Two typical adsorption sites, a bridge site at the edge of the NW and a hollow site on the facet of the NW, were examined on three surfaces, including (110), (310) and (2×1) missing-row reconstructed (MRR) (310).

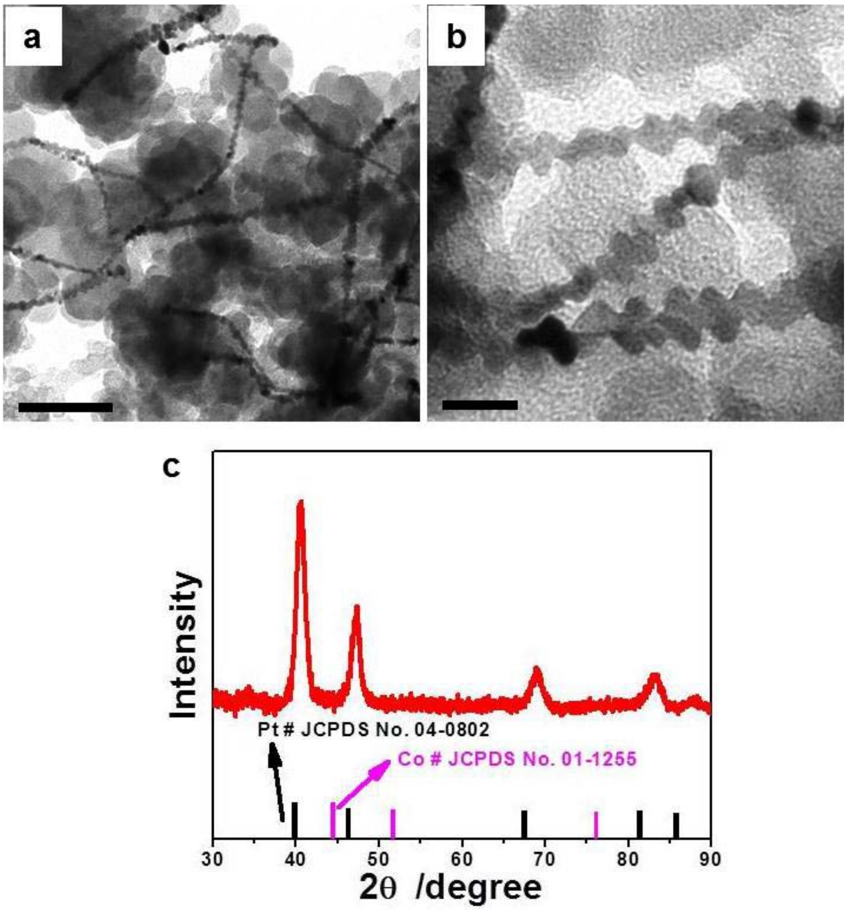

Supplementary Figure 26. Representative (a, b) TEM images and (c) PXRD pattern of hierarchical Pt_3Co/C after annealing at 500 ^{o}C for 1 h at N_2 atmosphere. The scale bars in (a) and (b) are 100 nm and 20 nm, respectively.

Supplementary Table 1. MOR activities of different catalysts. MOR measurements were performed at room temperature in 0.1 M $HClO_4$ + 0.2 M methanol solution at a scan rate of 50 mV s^{-1}.

Catalyst	Loading of metal Pt (μg)	Mass Activity at RT (A mg^{-1}_{Pt})	Specific Activity at RT (mA cm^{-2})
**Pt/C (JM)**	**1.25**	**0.256**	**0.44**
**$Pt_{10}Co$ NWs/C**	**1.25**	**0.232**	**0.90**
**Pt_7Co NWs/C**	**1.25**	**0.648**	**1.53**
**Pt_3Co NWs/C**	**1.25**	**1.016**	**1.95**
**Pt_3Co NPs/C**	**1.25**	**0.312**	**0.81**

Supplementary Table 2. EOR activities of different catalysts. EOR measurements were performed at room temperature in 0.1 M $HClO_4$ + 0.2 M ethanol solution at a scan rate of 50 mV s^{-1}.

Catalyst	Loading of metal Pt (μg)	Mass Activity at RT (A mg^{-1}_{Pt})	Specific Activity at RT (mA cm^{-2})
**Pt/C (JM)**	**1.25**	**0.240**	**0.41**
**$Pt_{10}Co$ NWs/C**	**1.25**	**0.336**	**1.30**
**Pt_7Co NWs/C**	**1.25**	**0.608**	**1.44**
**Pt_3Co NWs/C**	**1.25**	**0.808**	**1.55**
**Pt_3Co NPs/C**	**1.25**	**0.254**	**0.66**

Supplementary Table 3. ORR activities of different catalysts at 0.90 V *vs.* RHE. ORR measurements were performed at room temperature in O_2-saturated $HClO_4$ solution at a sweep rate of 10 mV s^{-1} and a rotation rate of 1600 rpm.

**Catalyst**	**Loading of metal Pt (μg)**	**Mass Activity at RT (A mg^{-1}_{Pt})**	**Specific Activity at RT (mA cm^{-2})**
**Pt/C (JM)**	**1.25**	**0.11**	**0.18**
**$Pt_{10}Co$ NWs/C**	**1.25**	**0.64**	**2.48**
**Pt_7Co NWs/C**	**1.25**	**2.77**	**6.54**
**Pt_3Co NWs/C**	**1.25**	**3.71**	**7.12**
**Pt_3Co NPs/C**	**1.25**	**0.53**	**1.36**
